# Targeted protein degradation: advances in drug discovery and clinical practice

**DOI:** 10.1038/s41392-024-02004-x

**Published:** 2024-11-06

**Authors:** Guangcai Zhong, Xiaoyu Chang, Weilin Xie, Xiangxiang Zhou

**Affiliations:** 1 Department of Hematology, Shandong Provincial Hospital Affiliated to Shandong First Medical University, Jinan, Shandong 250021 China; 2 Medical Science and Technology Innovation Center, Shandong First Medical University & Shandong Academy of Medical Sciences, Jinan, Shandong 250117 China; 3 School of Pharmaceutical Sciences, Pingyuan Laboratory, Zhengzhou University, Zhengzhou, 450001 China; 4 Institute of Materia Medica, Shandong First Medical University & Shandong Academy of Medical Sciences, Jinan, Shandong 250117 China; 5 Department of Hematology, Shandong Provincial Hospital, Shandong University, Jinan, Shandong 250021 China

**Keywords:** Drug development, Drug screening

## Abstract

Targeted protein degradation (TPD) represents a revolutionary therapeutic strategy in disease management, providing a stark contrast to traditional therapeutic approaches like small molecule inhibitors that primarily focus on inhibiting protein function. This advanced technology capitalizes on the cell’s intrinsic proteolytic systems, including the proteasome and lysosomal pathways, to selectively eliminate disease-causing proteins. TPD not only enhances the efficacy of treatments but also expands the scope of protein degradation applications. Despite its considerable potential, TPD faces challenges related to the properties of the drugs and their rational design. This review thoroughly explores the mechanisms and clinical advancements of TPD, from its initial conceptualization to practical implementation, with a particular focus on proteolysis-targeting chimeras and molecular glues. In addition, the review delves into emerging technologies and methodologies aimed at addressing these challenges and enhancing therapeutic efficacy. We also discuss the significant clinical trials and highlight the promising therapeutic outcomes associated with TPD drugs, illustrating their potential to transform the treatment landscape. Furthermore, the review considers the benefits of combining TPD with other therapies to enhance overall treatment effectiveness and overcome drug resistance. The future directions of TPD applications are also explored, presenting an optimistic perspective on further innovations. By offering a comprehensive overview of the current innovations and the challenges faced, this review assesses the transformative potential of TPD in revolutionizing drug development and disease management, setting the stage for a new era in medical therapy.

## Introduction

**Fig. 1:**
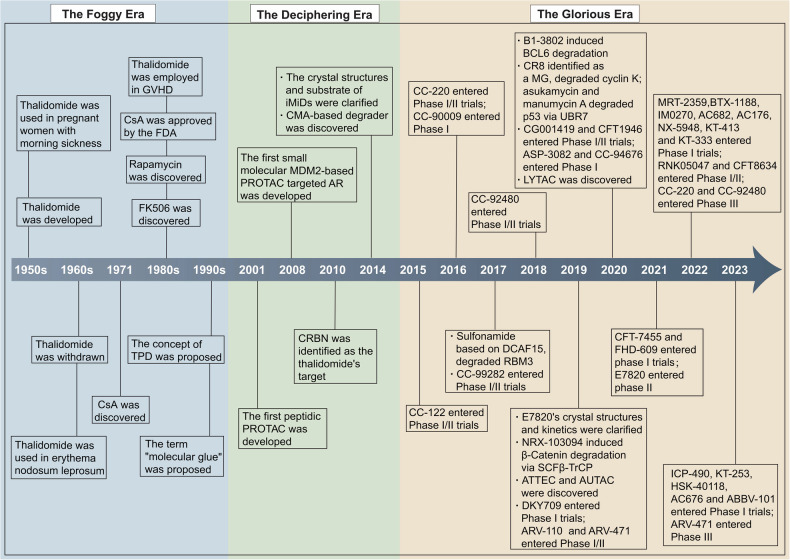
Timeline of the development of TPD technology. This timeline is divided into three pivotal eras: the Foggy Era, the Deciphering Era, and the Glorious Era. The Foggy Era is characterized by the initial development and subsequent withdrawal of Thalidomide, alongside the discovery of CsA, marking early applications of TPD under unclear mechanistic conditions. The Deciphering Era was marked by the formal establishment of the TPD concept and the elucidation of molecular mechanisms through the resolution of crystal structures. The Glorious Era has been distinguished by rapid clinical advancements, with several compounds progressing through various phases of clinical trials and the discovery of novel degradation pathways and mechanisms, such as LYTAC. This era highlights significant strides in the clinical application and understanding of TPD, potentially transforming treatment paradigms across multiple diseases

Despite chemotherapy remaining the primary cancer treatment, its efficacy is limited by response rate and inevitable drug toxicity. Over the past decades, remarkable advances have been made in the field of small molecule inhibitors (SMIs), which can more specifically target proteins of interest (POIs). For example, chronic myeloid leukemia (CML) has transitioned into a chemotherapy-independent chronic disease, markedly improving the 10-year survival rate to 83.3% through the application of tyrosine kinase inhibitors.^1^ However, challenges such as toxic side effects, drug resistance, and “undruggable” targets issue continue to persist. Low selectivity of drugs can inadvertently affect essential proteins, leading to off-target effects. Moreover, resistance may occur through various mechanisms, such as mutation, overexpression of the target POIs or adaptation to an alternative pathway.^2–4^ Furthermore, many potential proteins lack well-defined ligand-binding pockets, which makes them “undruggable” by conventional inhibitors.^5^ Targeted protein degradation (TPD) emerged as a promising strategy, utilizing intrinsic protein degradation systems, such as ubiquitin-proteasome system (UPS) and lysosome. It offers a valuable approach to potentially minimize off-target effects and overcome drug resistance,^6–11^ delivering targeted therapeutics for traditionally “undruggable” proteins,^12–16^ which were previously inaccessible through SMIs.^17^ The concept of TPD was formally introduced in 1999 by Proteinix through a patent application,^18^ transitioning from a “foggy era” where the mechanisms of protein degraders were poorly understood. Subsequently, the focus shifted towards elucidating the molecular mechanisms of these agents, marking the beginning of the “deciphering era”. This period is characterized by the development of proteolysis targeting chimeras (PROTACs)^19^ and a clearer understanding of the mechanisms of molecular glues (MGs).^20^ Building on these insights, TPD has now entered the “glorious era”, characterized by an explosion in research that has developed novel MGs and PROTACs. Many of them have entered clinical trials (Fig. 1). Over the past five years, lysosome-based TPD has emerged, broadening the substrate spectrum that encompasses the degradation of extracellular proteins. Recent literature has begun to elucidate this development.^21,22^ In this review, we explore the development and optimization of TPD, especially PROTACs and MGs, to underscore their transformative potential and efforts to boost their effectiveness and clinical use, paving the way for a “fruitful era”.

## Different TPD strategies: mechanisms, development, and advancement

### Proteasome-based degradation

**Fig. 2:**
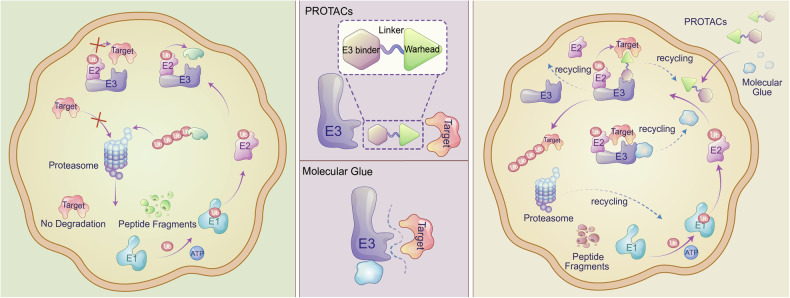
Schematic overview of TPD modalities. The left panel displays the ubiquitin-proteasome system and a protein not specifically targeted by E3 ubiquitin ligases, remaining undegraded. The middle panel presents the structural configurations of molecular glues (MGs) and proteolysis-targeting chimeras (PROTACs). The right panel details the TPD process, illustrating the cyclic interaction of PROTACs with a target protein and an E3 ligase, as well as the role of MGs in facilitating protein–protein interactions between E3 ligases and target proteins, thereby enhancing their association. This culminates in the ubiquitination and proteasomal degradation of the target protein, after which those modulators molecules are recycled

Among the leading innovations in TPD are PROTACs and MGs, which promote protein degradation via the UPS, a pathway that tags proteins for breakdown via enzyme cascades. Ubiquitinated proteins are then processed by the proteasome, with ubiquitin chains recycled by deubiquitinating enzymes^23,24^ (Fig. 2). Despite utilizing the same system, they operate via distinct mechanisms and exhibit unique characteristics (Table 1).

**Table 1:** Key differences between PROTAC and MG

Feature	PROTAC	Molecular Glue
Molecular weight	Larger, often >700 Da	Smaller, typically <500 Da
Structure	Biovalent	Monovalent
Mechanism	Recruit target proteins to an E3 ligase	Induce PPIs
Bioavailability	Worse	Better
Discovery	Rational design	Serendipitous
Development	Rational design	High-throughput screening
Routes of administration	Injection preferred; oral formulations are being developed	Potential for oral administration
Concentration window	Narrower due to hook effect	Wider
Representative E3 ligases	CRBN, VHL, IAP and MDM2	CRBN and DCAF15
Representative targets	AR, ER and BTK	IKZF1 and IKZF3

*CRBN* cereblon, *VHL* Von Hippel-Lindau, *IAP* an inhibitor of apoptosis protein, *MDM2* minute 2 homolog, *AR* androgen receptor, *ER* estrogen receptors, BTK Bruton's tyrosine kinase, *PPIs* protein–protein interactions, *DCAF15* DDB1 and CUL4 associated factor 15, *IKZF1/3* IKAROS family zinc finger 1/3

PROTACs function in a ternary complex, which is composed of a POI-targeting ligand, an E3 ligase ligand, and a linker. This method allows for the specific degradation of various proteins and the recyclability of PROTACs, increasing their efficacy. However, the hook effect can occur at high concentrations, disrupting ternary complex formation and reducing efficacy.^25^ Moreover, PROTACs have encountered challenges, primarily due to their large size and complex structure, which can reduce their stability and cellular permeability.^26,27^ Current efforts are aimed at designing smaller, more stable PROTACs to improve their druggability and bioavailability.^28,29^ Conversely, MGs, are small molecules that modulate protein–protein interactions (PPIs) to facilitate the degradation of POIs by promoting interaction between an E3 ligase and the target protein. Their smaller size and simplicity offer advantages such as better cellular permeability and potential oral administration. However, designing effective MGs is complex due to the unpredictable nature of PPIs, with many discovered serendipitously. Unlike PROTACs, MGs do not experience the hook effect. Their development often relies on innovative screening methods to identify compounds capable of effectively modulating PPIs.^30–33^ Both PROTACs and MGs complement each other, advancing the potential of TPD techniques in disease treatment. Addressing the unique challenges of each could further enhance their efficacy and clinical applicability.

#### PROTACs

The first PROTAC based on peptidic backbones, was reported by Sakamoto et al., which faced limitations due to poor cell permeability from its high molecular weight.^26^ In 2008, small-molecule PROTACs were synthesized to enhance cellular uptake and pharmacokinetics.^29^ Building upon this, the amount and efficacy of PROTACs have significantly increased, with many advancing into clinical trials. The protein degradation potential of PROTACs varies with factors such as ligands interactions with POIs and E3 ligases, linker length and composition, as well as the cellular milieu. Ongoing research efforts are dedicated to enhancing the degradation efficacy of PROTACs and improving drug characteristics like bioavailability.

##### E3 ligases

E3 ligases represent an attractive intervention point within the UPS. These enzymes facilitate the ubiquitin molecules from E2 to specific target proteins thereby orchestrating the ubiquitination process. The human genome encodes over 600 E3 ligases,^34^ yet only a few dozen ligands for these enzymes are applied in TPD,^35^ focusing mainly on four major E3 ligases: the mouse double minute 2 homolog (MDM2), an inhibitor of apoptosis protein (IAP), von hippel-lindau (VHL) and cereblon (CRBN). Discovering novel E3 ubiquitin ligases and optimizing their ligands are critical for enhancing drug properties and pharmacological efficacy.

*MDM2*: MDM2, an E3 ubiquitin ligase, can inhibit the tumor-suppressor functions of p53.^36^ Over the past two decades, numerous SMIs designed to disrupt the p53-MDM2 interaction have been developed,^37^ but challenges like toxicity and resistance—often due to TP53 gene mutations—limit their efficacy.^38,39^ The first small molecule PROTAC based on MDM2 inhibitor Nutlin-3, showed modest capability in degrading the androgen receptor (AR).^29^ Subsequent PROTAC design based on Nutlin-3 have improved degradation of POIs and induced significant cytotoxic effects in cancer cells without adversely affecting normal cells.^40^ Furthermore, MDM2-recruiting PROTACs have stabilized p53,^41^ leading to significant anti-proliferative effects in certain myeloid leukemia cells. The development of homo-PROTAC employing two MDM2 ligands, initiated the self-degradation of MDM2 in A549 cell line, providing initial evidence of its effectiveness in vivo.^42^ One promising MDM2-based PROTACs, KT-253, outperforming traditional MDM2 inhibitors by more than 200-fold. This increase in potency has been validated through sustained tumor regression observed in xenograft models.^43^ Consequently, KT-253 has entered a Phase I clinical trial. Despite the promising prospects of MDM2-based PROTACs in inducing apoptosis in p53 wild-type tumor cells, challenges such as complex synthesis, high molecular weight, and lipophilicity continue to impede their broader development.^27^

*IAPs*: Apart from as E3 ubiquitin ligases, IAPs also function as suppressors of apoptosis by blocking caspase.^44^ Among the IAP family, c-IAP1, c-IAP2, and XIAP are regarded as potential effective targets for cancer therapy due to their overexpression in cancer cells^45^ and roles in anti-apoptosis. Numerous potent SMIs targeting IAPs have been developed^46^ and further utilized as ligands in PROTACs. These PROTACs, known as specific and nongenetic IAP-based protein erasers (SNIPERs), have shown effectiveness in simultaneous knocking down the POI and cIAP1, expanding the potential for targeting a diverse range of proteins for degradation. Among the popular ligands for IAPs, LCL-161 takes the lead, closely followed by bestatin and MV1 derivatives.^47^ Bestatin acts as an inhibitor of cIAP, while LCL-161 and MV1 act as pan antagonists, targeting both c-IAP and XIAP. Methyl bestatin was used to synthesize the first SNIPERs in 2010,^48^ which degraded POI at high concentrations and induced autoubiquitination of cIAP1. Natio’s group further modified methyl bestatin to create amide-type SNIPERs for more selective knockdown of POIs.^49^ In 2012, MV1-based SNIPERs capable of dual degradation of both the POI and IAPs, exhibited stronger anti-proliferative activity.^50^ In subsequent studies, SNIPERs with pan antagonists like MV1 and LCL-161, displayed greater efficiency than bestatin-based compounds.^51^ SNIPERs possess the unique feature of simultaneous knockdown of the POI and cIAP1, making them valuable tools for the degradation of a variety of POIs.

*VHL*: VHL acts as a substrate recognition subunit of the E3 ligase complex, specifically targeting hypoxia-inducible factor 1α (HIF-1α) under normoxic conditions through UPS.^52^ HIF-1α, a transcription factor, primarily manages response to hypoxia, regulating processes such as erythropoietin synthesis, angiogenesis suppression, and cancer metastasis.^53–55^ The development of VHL ligands was guided by the structural analysis of VHL-HIF-1α interaction. Recent advancements have included the creation of PROTACs with these ligands, significantly enhancing their degradation efficacy at the cellular level. The first small molecule VHL ligand was designed through modifying a small peptide fragment of HIF-1α,^56^ according to the co-crystal structure of VHL bound to HIF-1α.^57,58^ The most potent ligand was further modified to generate ligand 51, displaying high affinity, moderate potency and limited cell permeability.^59^ In 2014 and 2017, optimized SMIs, VH032^60^ and VH298,^61^ were discovered, respectively, with nanomolar binding affinity for VHL. The development paved the way for the creation of the first PROTAC based on VH032 in 2015, exhibiting a remarkable 90% degradation of POI at the cellular level.^28^ Remarkably, due to the limited presence of VHL in platelets, VHL-based PROTACs hold great potential as an alternative therapeutic approach for mitigating platelet-related toxicity.^62^

*CRBN*: CRBN, another critical substrate receptor of the CRL4 E3 ligase complex, is targeted by thalidomide and its analogs, also known as immunomodulatory drugs (IMiDs) like pomalidomide and lenalidomide. These drugs bind to CRBN, leading to the CRBN-dependent degradation of substrates such as IKAROS family zinc finger proteins 1 and 3 (IKZF1/3), pivotal in disease-related protein degradation.^63–66^ Thalidomide gained infamy due to its teratogenic effects but has been repurposed in PROTAC technology for targeted protein degradation. In 2015, the first CRBN-recruiting PROTAC, dBET1, was generated,^67^ following the report of co-crystal structure of DDB1–CRBN–thalidomide complexes.^68,69^ dBET1 exhibited pronounced depletion ability of POIs at 100 nM in acute myeloid leukemia (AML) cell lines. Given the satisfactory clinical effectiveness and low molecular weight of IMiDs, many labs dedicated to explore novel CRBN modulators. Of note, TD-106, a pomalidomide derivative, was used to synthesize potent bromodomain-containing protein 4 (BRD4) and AR PROTACs.^70,71^ While IMiDs were prone to hydrolysis,^67,72^ this problem was effectively addressed by the development of phenyl-glutarimide analogs-based PROTACs, which maintained targeting specificity without degrading IKZF1/3.^73^ Another novel CRBN ligand, phenyl dihydrouracil, enhanced binding affinity, leading to highly potent degradation with low cytotoxicity.^74^ Recently, achiral phenyl dihydrouracil (also called PDHU) derivatives were designed as CRBN ligands and corresponding PROTACs exhibited robust degradation at picomolar concentrations.

Due to their stable metabolism, potent degradation capabilities, broad distribution, and relatively low molecular size, CRBN ligands are increasingly favored in the development of orally bioavailable PROTACs currently advancing in clinical trials.

*Others*: Researchers have uncovered that expression levels and types of E3 ligases vary across tissues, which affect the degradation activity of PROTACs.^75–78^ Moreover, acquired resistance to PROTACs has been linked to genomic alterations in the core components of E3 ligases.^79^ To expand the spectrum of degradable targets, ongoing studies are focused on identifying and utilizing a wider range E3 ligases. Beyond these four common E3 ligase ligands mentioned above, more than a dozen additional E3 ligases^80–98^ have been identified and harnessed in the development of PROTACs, as descripted in Table 2.

**Table 2:** The additional E3 ligases for PROTACs

E3 ligase	Compound	Ligand	Targets
AhR^80^	β-NF-JQ1; α-NF-JQ1; ITE-ATRA	β-NF	BRD2/3/4
FEM1B^81^	NJH-1-106	EN106	BRD4; BCR-ABL
KEAP1^82–86^	CDDO–JQ1; 955; PL-ceritinib conjugate; MS83; DGY-06-177-pk2; Peptide 1;	CDDO; KEAP1-L; KI696; Ac-LDPETGEYL-OH; bardoxolone methyl; Piperlongumine	BRD3/4; CDK9; EML4-ALK; FAK; Tau
RNF4^87^	CCW 28-3	JQ1	BRD4
RNF114^88–90^	XH2; ML 2-14; ML 2-22; ML 2-23	Nimbolide; EN219	BRD4; BCR-ABL
L3MBTL3^91^	KL-4	UNC1215	FKBP12; BRD2
DCAF1^92^	YT41R; YT47R	MY-11B	FKBP12; BRD4
DCAF11^93^	21-SLF; 21-ARL; 10-SLF	21-SLF	FKBP 12; AR
DCAF15^94,95^	Undefined; DP1	Indisulam; E7820	BRD4/7/9
DCAF16^96,97^	C-KB02-SLF; C8	KB02; KB03; KB05	FKBP12; PARP2
KLHL20^98^	BTR2003	BTR2000	BRD2/3

*AhR* aryl hydrocarbon receptor, *FEM1B* feminization 1 homolog B, *KEAP1* Kelch-like ECH-associated protein 1, *RNF* ring finger protein, *L3MBTL3* Lethal(3)malignant brain tumor-like protein 3, *DCAF* DDB1 and CUL4 associated factor, *BRD* BET bromodomain protein, *CDK9* cyclin-dependent kinase 9, *EML4-ALK* echinoderm microtubule-associated protein-like 4-anaplastic lymphoma kinase, *FAK* focal adhesion kinase, *FKBP* FK506-binding protein, *AR* androgen receptor, *PARP2* Poly(ADP-ribose) polymerase 2, *KEAP1* Kelch-like ECH-associated protein 1*, KLHL 20* Kelch-like protein 20

##### Linker

In ubiquitination-mediated degradation, the linker is essential for formation of POI-PROTAC-E3 ternary complex, influencing PROTACs’ efficacy and specificity^99,100^ through its attachment points and the chemical properties such as length^101–103^ and flexibility. Innovations in linker design, informed by co-crystal structures and computational modeling, have encouraged the identification of optimal attachment points and appropriate linker lengths.^100^ Not surprisingly, the structure and physical properties of chemical groups in the linker are crucial for optimizing PROTAC molecules. Commonly used in linkers, polyethylene glycol (PEG) and alkane chains provide adjustable lengths and compositions, facilitating flexibility and a hydrophobic collapse that enhances permeability and solubility.^104–106^ These linkers are often employed in PROTAC designed as tool molecules for research. In contrast, rigid linkers demonstrate greater stability than flexible linkers. After determining the optimal linker length using PEG and alkane, similar-length rigid linkers can be introduced as alternatives to enhance the solubility, bioavailability, and even the degradation potency of PROTACs.^107^ Interestingly, Ciulli’s group designed a macrocyclic linker for PROTAC MZ1 by placing a second linker between the VHL ligand and the first PEG linker to stabilize the bioactive conformation. Compared to MZ1, macroPROTAC-1 demonstrated lower binding affinity but similar cellular potency.^108^

#### MGs

MGs initially garnered attention for their unique ability to stabilize or induce PPIs without natural affinity between the proteins.^109–112^ Subsequent research demonstrated that MGs could effectively facilitate the formation of complexes between E3 ligases and target proteins, leading to protein degradation.^63,66,68,69,113,114^ Unlike PROTACs, MGs are smaller, monomeric molecules that generally adhere to Lipinski’s Rule of Five, suggesting superior drug-like properties such as enhanced oral bioavailability and favorable pharmacokinetics. Despite these advantages, the discovery of MGs remains challenging, heavily reliant on the identification of natural or incidental interaction sites. To date, only three amide-based MGs have been approved for oral use in multiple myeloma (MM) and myelodysplastic syndromes (MDS).^114,115^ The burgeoning interest in this field has prompted substantial investment, with several MGs now advancing through clinical trials.

##### Non-degradative MGs

Early and notable examples of MGs include microbial macrolides such as FK506, rapamycin and the cyclosporin A. Discovered in 1971, cyclosporin was first noted for its immunosuppressive properties by a Swiss biologist at Sandoz. Both FK506 and rapamycin, known for their cyclosporin A-like activities, share a large cyclic polyketide structure. In the following years, scientists raced to uncover their mechanisms of action. By the early 1990s, it was discovered that cyclophilin and FKBP12 were the respective receptors for cyclosporin A and FK506, forming complexes that inhibit the protein phosphatase calcineurin and exert immunosuppressive effects. Interestingly, calcineurin can only bind to complexes, but not the free cyclophilin or FKBP. MGs act as an adhesive that facilitates PPIs between proteins that naturally do not possess affinity for each other. The term “molecular glue” was coined in 1992 to describe its mode of action.^116^ In 1994, research revealed that FKBP12-rapamycin interacts with the protein kinase mTOR.^117,118^ Mutations in mTOR or FKBP12 lead to rapamycin resistance, validating the notion of rapamycin as a MG.^119,120^ By the end of the 20th century, all three drugs had been approved by FDA for preventing organ transplant rejection.

##### Degradative MGs

IMiDs, such as thalidomide, lenalidomide, and pomalidomide, are key examples of degradative MGs used in therapy. Originally marketed as a sedative, thalidomide was later linked to severe teratogenic effects, leading to its withdrawal. The rediscovery of thalidomide’s benefits in treating leprosy sparked renewed interest, resulting in the development of more potent analogs such as lenalidomide and pomalidomide, which showed promising anti-inflammatory^121,122^ and anti-angiogenic efficacy.^123,124^ These new discoveries promoted broader application of IMiDs against various hematological malignancies, such as MM, MDS, chronic lymphocytic leukemia (CLL) and B-cell lymphoma.^114,125–129^ A breakthrough came in 2010 when thalidomide was found to target the E3 ubiquitin ligase CRBN, elucidating the molecular basis of its effects.^65^ This discovery advanced lenalidomide as a MG that enables CRBN to target specific proteins for degradation, including the lymphoid transcription factors IKZF1 and IKZF3^65,68^ and CK1α in MDS with deletion 5q.^114^ The elucidation of the mechanisms by which IMiDs operate has sparked significant interest in developing new MGs. The discovery of the arylsulfonamide-based drugs, such as indisulam and E7820, further expanded the scope of MGs. In 2017, Han et al. discovered that indisulam promotes the recruitment of RNA-binding motif protein 39 (RBM39) to the CUL4-DCAF15 E3 ligase complex for degradation.^130^ Early Phase II clinical trials have shown limited efficacy. To date, arylsulfonamide have not yet got regulatory approval.^131^ Moreover, Słabicki et al. found that BI-3802, an inhibitor of BCL-6, functioned as a MG by promoting the oligomerization of BCL6 and facilitating its interaction with SIAH1, an E3 ligase. This interaction enhances the ubiquitination and subsequent degradation of BCL6, showcasing the potential of MGs in disease therapy.^132^

Recent research has moved away from the incidental repositioning of existing drugs for new therapeutic applications and focused on the targeted discovery of MGs instead. Słabicki and colleagues embarked on a comprehensive screening, assessing the cytotoxicity of 4518 SMIs across 499 cancer cell lines to identify potential E3 ligase targets. Their research unveiled CR8, a cyclin-dependent kinase (CDK) inhibitor, as an MG that orchestrates the formation of a complex between CDK12-cyclin K and DDB1, an adapter protein of the E3 ligase CUL4. This complex formation triggers the ubiquitination and degradation of cyclin K, exerting a profound antitumor effect.^133^

Furthermore, advancements in chemoproteomics and the exploration of polyvalent natural products have opened new avenues for MG discovery. For example, natural polyketide manumycins, asukamycin, and manumycin A, have been found to mediate the tumor suppressor function of TP53 through MG-like interactions with UBR7.^134^ This interaction underscores the potential of chemically diverse substances to reveal unique mechanisms of action, providing a strategic pathway for the discovery of novel therapeutic agents.

Despite these advancements, the discovery of MGs faces significant challenges, primarily due to the reliance on retrospective elucidation of action modes and the serendipitous nature of such discoveries. To date, only a handful of MGs have been successfully identified and developed into therapeutic agents, highlighting the need for more systematic and targeted approaches in MG research. The application of advanced screening methodologies and discoveries of complex biological interaction pattern remain crucial for overcoming these challenges and enhancing the therapeutic arsenal available for treating various diseases.

### Lysosome-based degradation

While the UPS remains fundamental to TPD technologies, the lysosome significantly broadens these approaches.^135,136^ Lysosome facilitates the breakdown of a diverse range of cellular constituents, including persistent proteins, aggregates, nucleic acids, lipids, organelles, and intracellular parasites, via mechanisms like endocytosis and autophagy.^135,137,138^

#### The protein degradation mechanism of lysosome

Lysosomes are membrane-enclosed cytoplasmic organelles, which contain more than 60 hydrolytic enzymes including proteases, nucleases, glycosidases, lipases, phospholipases, phosphatases and sulfatases.^139,140^ These enzymes, all acid hydrolases, are active at the acidic pH about 4.5.^141^ To maintain the acidic environment, lysosomal membrane contains vacuolar H^+^-ATPases (V-ATPases).^142,143^ Lysosomes have a broader degradation capability than proteasomes, including both from inside cell and taken from outside.^144,145^ As the cellular waste disposal system, lysosomes degrade components taken up from the outside through endocytosis and those from inside the cell through autophagy.^146^ Lysosome-based degradation technology is designed by utilizing both autophagy and endocytosis.

##### Autophagy

The autophagy-lysosomal pathway is a conserved mechanism for degradation,^147,148^ playing a critical role in cellular differentiation, defense, growth regulation, tissue remodeling, acclimatization and so on. As a major intracellular degradation system, autophagy ultimately directs materials to be degraded in the lysosome.^149^ Targeting the autophagy-lysosome system has emerged as a promising therapy for diseases treatment, such as neurodegenerative disorders and cancer.^150,151^ There are three distinct pathways to the lysosome, including macroautophagy, chaperone-mediated autophagy (CMA) and microautophagy.^148^ Among these three pathways, lysosome-based degradation technology promotes only relied on macoautophagy and CMA pathway.

*Macroautophagy*: During the macroautophagy process, autophagosomes which are double-membrane-bound vacuoles, form and capture cytoplasmic cargo to deliver them to lysosomes.^152,153^ Then autophagosome membrane could fuse with the lysosome, facilitating cargo degradation by lysosomal hydrolases. Macroautophagy can be divided into nonselective and selective process.^152^ In the nonselective process, random cytoplasm is isolated by autophagosomes. In contrast, selective macroautophagy involves specific cargos that are recognized and regulated by the cargo receptor proteins on the membrane.^152,154^

The autophagosome is an essential double-membrane vesicle for macroautophagy.^155,156^ They are formed through the action of more than 30 autophagy-related genes (ATG), initially identified in yeast,^157^ many of which have mammalian orthologs. Among these genes, 18 different ATG proteins are involved in autophagosome formation.^158^ In mammalian cells, autophagosome induction is regulated by ULK1/2 (unc-51 like kinase), Atg13, FIP200 (200 kDa focal adhesion kinase family-interacting protein) and Atg101.^159,160^ The formation of autophagosomes is supported by Atg9/ATG9A vesicles, serving as the membrane source. The vesicle nucleation is regulated by the class III phosphatidylinositol 3-kinase (PtdIns3K) complex including PIK3C3/VPS34, PIK3R4/VPS15, Beclin 1, and Atg14,^159,161,162^ which facilitates the recruitment of PtdIns3P-binding proteins such as WIPI. The Atg2-Atg18/WIPI complexes can mediate phagophore membrane expansion.^163,164^ In addition, two ubiquitin-like conjugation systems, ATG8-family protein members and Atg12-Atg5 complex mediate the autophagosome mature.^158^

Selective macroautophagy is mediated by selective autophagy receptors (SAR),^165^ which attach to cargoes and interact with the autophagosome membrane protein by Atg8/LC3 interacting region (LIR).^166,167^ Atg8, a ubiquitin-like protein, is crucial for the formation of autophagosomal membranes. In mammalian cells, there are 7 homologs of Atg8 such as GABARAP, and various forms of MAP1LC3. SARs like p62/SQSTM1 can interact with LC3, initiating cargo degradation.^168^

*Aggrephagy*: Aggrephagy involves the degradation of misfolded or aggregated proteins. Initially, protein aggregates are ubiquitinated by E3 ligase Parkin, forming aggresomes. These are subsequently recognized by SARs such as p62/SQSTM1 (Sequestosome-1), neighbor of BRCA1 gene 1 (NBR1), and optineurin (OPTN).^169,170^

*Mitophagy*: Damaged or excess mitochondria is degraded through mitophagy, including ubiquitin-dependent and ubiquitin-independent pathways. The ubiquitin-dependent PINK1-Parkin pathway degrade heavily depolarized mitochondria, involving essential components including NDP52, p62, TAX1BP1, AMBRA1, and OPTN.^171,172^ Ubiquitin-independent mitophagy, critical for mitochondria homeostasis, relies on receptors such as BNIP3L/NIX, NLRX1, AMBRA1, BNIP3, FUNDC1 and FKBP8.^171,173^

*Lysophagy*: Lysophagy is crucial for maintaining lysosomal homeostasis, including the removal of damaged or excess lysosomes. Lysophagy is essential and ubiquitination regulated by ubiquitination from p62 and TRIM16.^148,174^

*Pexophagy*: Pexophagy targets and degrades peroxisomes, which are involved in oxidative reactions and lipid metabolism, to maintain peroxisome homeostasis. This process is associated with p62 and NBR1.^175,176^

*Xenophagy*: Xenophagy targets and eliminates intracellular pathogens, including bacteria, viruses, parasite and fungi. Key SARs in xenophagy include NDP52, TAX1BP1, OPTN, p62.^177,178^

*Endoplasmic reticulum (ER)-phagy*: ER plays crucial roles in various cellular processes, including calcium storage, protein synthesis, folding, modification, transport, and lipid metabolism. It’s so critical for transportation system that breaking ER homeostasis associated with various diseases, such as Alzheimer’s disease, Crohn’s disease and some neurodegenerative diseases.^179,180^ ER-phagy, and ER-associated degradation help to rebuild ER homeostasis by the degradation of damaged and excess ER subdomains. Up to now, there are six receptors related to ER-phagy are found, namely FAM134B, SEC62, RTN3, CCPG1, ATL3, TEX264.^181,182^

*Chaperone-mediated autophagy (CMA)*: CMA is another type of autophagy that depends on the chaperone proteins to selectively transport cytosolic proteins directly across the lysosomal membrane for degradation.^183^ Unlike yeast, which is essential for studying macroautophagy and microautophagy, CMA is exclusive to birds and mammals.^184^

Sharing the similar group of substrate proteins, CMA and eMI selectively degrade protein with KFERQ-like motif, which can bind protein HSC70 then delivers it to the membrane of lysosomes.^185^ The residue sequence of the motif is variable, allowing for residue substitution with similar properties, thus maintaining recognition by HSC70.^183,186^ Typically, the motif is bracketed by a glutamine (Q) residue and comprises one acidic (either glutamic acid (E) or aspartic acid (D)), one or two basic (lysine (K) or arginine (R)), and one or two hydrophobic residues (phenylalanine (F), valine (V), leucine (L), or isoleucine (I)). Post-translational modifications, such as phosphorylation or acetylation, can alter the charge of these residues, enhancing motif recognition even when incomplete.^187,188^

In the CMA pathway, HSC70 acts as a molecular chaperone, guiding the proteins with the KFERQ-like motif across the lysosomal membrane. This process requires several co-chaperones, such as HSP40, HSC70-interacting protein, HSP70-HSP90 organizing protein (HOP), and Bcl2-associated athanogene-1 (BAG-1), which assist in the translocation but do not interact directly with the motif.^189,190^

Upon reaching the lysosomal membrane, the substrate protein complex binds to lysosome-associated membrane protein type 2 A (LAMP2A), a splicing variant of the LAMP2 gene exclusive to birds and mammals.^191^ LAMP2A is crucial for CMA, distinguishing it from eMI, as it facilitates the direct translocation of unfolded substrate proteins through the lysosomal membrane, unlike in eMI where protein unfolding and LAMP2A are not required.^192^ LAMP2A initially functions as a monomer. Upon activation, it forms a 700 kDa homotrimer with the assistance of luminal Hsp90, enabling substrate translocation.^193^ This complex rapidly disassembles back into monomers post-translocation.^185,194^ Approximately 40% of proteins in the mammalian proteome contain a KFERQ-like motif,^195^ highlighting the selectivity and critical role of CMA in regulating cytosolic signaling pathways associated with cancer and neurodegenerative disorders.^196^

##### Endocytosis

Unlike autophagy, endocytosis is a cellular process associated with internalization substances from extracellular.^197^ It refers to another pathway of lysosome-based degradation technology. This process involves the invagination of the plasma membrane, forming a vesicle that encases the ingested substances.^198^ Endocytosis plays a critical role in various physiological processes.^197,199^ There are six types of endocytosis, each characterized by distinct mechanisms: phagocytosis,^200^ pinocytosis,^201^ receptor-mediated endocytosis (RME),^202^ caveolae-mediated endocytosis,^203^ clathrin-independent carriers (CLIC) and the glycosylphosphatidylinositol-anchored proteins-enriched early endosomal compartment (GEEC) endocytosis^204^ and fast endophilin-mediated endocytosis (FEME).^205^

*Phagocytosis*: Phagocytosis involves cells such as macrophages and dendritic cells ingesting particles larger than 0.5μm, including pathogens and dead cells.^206^ These particles are sequestered into phagosomes formed from the plasma membrane and transported to lysosomes for degradation. The process is regulated by actin remodeling via Cdc42 and RAC1^207^ and vesicle scission is mediated by the GTPase dynamin.^208^ Key receptors in phagocytosis include Fc receptors, complement receptors, α5β1 integrin, Dectin 1, MARCO, scavenger receptor A, and toll-like receptors.^209–215^

*Pinocytosis*: Pinocytosis, also termed ‘cell drinking’, involves the uptake of small molecules dissolved in extracellular fluids. The process starts with cell surface ruffling and the formation of a vesicle by the invaginating plasma membrane.^216^ This vesicle, known as a macropinosome, is dynamin-independent and can merge with a lysosome to create a macropino-lysosome.^217^ Pinocytosis facilitates cell motility, antigen presentation to T cells, and nutrient uptake. It is categorized by vesicle size into micropinocytosis (~0.1 μm diameter) and macropinocytosis (0.5–5 μm diameter).^218^ Various growth factors, including CSF-1, EGF, and PDGF, stimulate pinocytosis.^219–221^ The process is regulated by proteins such as Ras, PI3-kinase, Rab5, Rabankyrin-5, Rac, Cdc42, PAK1, CtBP1/BARS, SWAP-70, and SNX family members.^197,222–224^

*RME*: RME, also known as CME, is a clathrin and dynamin-dependent process. It is primarily driven by surface receptors that can bind to their ligands, initiating cargo recruitment.^225^ The process begins with the recruitment of endocytic proteins at the plasma membrane, mediated by phosphatidylinositol 4,5-bisphosphate (PI(4,5)P2).^226^ These proteins assemble into a clathrin coat composed of clathrin, adapter proteins such as AP2 and CALM, and scaffold proteins including EPS15 and EPS15R.^225–227^ During clathrin coat formation, cargoes are recruited to the plasma membrane. The membrane bending necessary for vesicle formation is facilitated by the actin cytoskeleton, with proteins like the Wiskott-Aldrich syndrome protein family initiating actin filament formation.^228^ After membrane bending, membrane fission and clathrin-coated vesicle scission is launched, during this process, dynamin assembles the ‘nick’ of clathrin-coated pit.^225^ Dynamin then mediates membrane fission, a process driven by GTP hydrolysis. BAR domain proteins, differing in curvature, aid in dynamin recruitment and vesicle membrane formation. Finally, the clathrin coat is disassembled by auxilin and HSC70, following the dephosphorylation of PI(4,5)P2 to phosphoinositol 4-phosphate (PI4P).^225,229,230^ This intricate system involves over 50 proteins and has significant implications for drug design targeting CME pathways.

*Caveolae-mediated endocytosis*: The caveolae-mediated endocytosis is clathrin-independent type endocytosis, characterized by plasma membrane invaginations with a diameter of ~50–100 nm. The unique membrane composition of caveolae includes glycosphingolipids and cholesterol, with caveolin-1 being the principal structural protein.^203^ The caveolae membrane also incorporates GPI-anchored proteins (GPI-AP), various receptors, and non-receptor protein tyrosine kinases.^231^ ATPase EH domain-containing protein 2 stabilizes caveolae on the cell surface.^232^ This endocytic pathway plays roles in cell signaling, lipid regulation, and pathogen entry. Substances internalized via caveolae range from small molecules and proteins such as folic acid, albumin, and interleukin-2 (IL2); to toxins and viruses including cholera toxin, tetanus toxin, Simian Virus 40, polyoma virus, Echovirus 1, and FimH-expressing *E. coli*.^203,233,234^

*CLIC/GEEC endocytosis*: CLIC/GEEC endocytosis is a clathrin-independent pathway involving uncoated tubular carriers known as CLICs, which evolve into tubular endocytic compartments called GEECs.^197^ Originating from the plasma membrane, the invagination process in CLIC/GEEC endocytosis is mediated by galectin-3, which binds to glycosylated proteins at the membrane. This pathway primarily facilitates the selective internalization of GPI-AP and also transports glycosylated transmembrane proteins such as CD44 and CD98, along with cholera toxin B-subunit (CTxB).^204,235^ Regulatory proteins for this endocytosis include Cdc42 and ADP ribosylation factor (ARF1).^236^ While dynamin was not initially considered a mediator in the CLIC/GEEC pathway, recent studies have shown that dynamin function is crucial, as its acute inhibition can significantly impact CLIC/GEEC endocytosis.^235,237^

*FEME*: FEME is a clathrin-independent but dynamin-dependent pathway, activated rapidly by receptor-ligand interactions, primarily transporting receptors.^197,238^ Before receptor activation, FEME necessitates the pre-enrichment of endophilin A2 into discrete clusters on the plasma membrane, as they are governed by endophilins A1, A2, and A3, all of which are BAR domain proteins, with endophilin A2 being the predominant regulator.^239^ The recruitment of the complex including FBP17 and CIP4 to Pi(3,4,5)P3 patches leads to clustering of the 5′-phosphatases SHIP1/2 and Lamellipodin, which in turn recruits and enriches endophilin A2.^204,239^

Following this, endophilin A2 regulates the formation of FEME carriers, allowing rapid receptor activation. Should the receptor remain inactive, proteins such as GTPase-activating proteins RICH1, SH3BP1, and Oligophrenin quickly disassemble the endophilin A2-enriched complex.^204,239–241^ Unlike other endocytic processes, FEME is highly specific, with each vesicle typically transporting only one type of cargo determined by the receptor species.

**Fig. 3:**
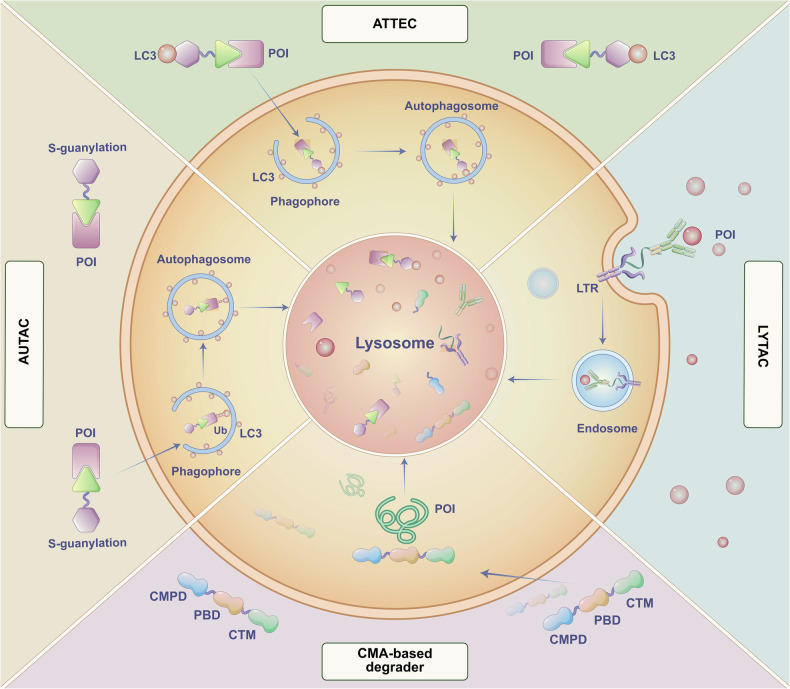
Lysosome-dependent protein degradation strategies. AUTAC, ATTEC, CMA-based degraders and LYTAC. AUTAC, ATTEC promote POI degradation through macroautophagy involved in autophagosome formation. CMA-based degraders promote POI degradation through chaperone-mediated autophagy. LYTAC promotes POI degradation through endocytosis involving endosome formation. POI protein of interest, LTR lysosome-targeting receptor, CTM CMA-targeting motif, PBD protein binding domain, CMPD cell membrane penetration domain

In the broader context, lysosome-based degradation pathways demonstrate extensive capabilities for degrading long-lived proteins and aggregates, enhancing the range of degradation targets and techniques beyond those available through proteasome-based degradation. By leveraging mechanisms from endocytosis and autophagy, innovative strategies can be developed to regulate lysosomal uptake and design new methods for degrading specific proteins via different lysosomal degradation pathways (Fig. 3).

#### Novel lysosomal targeting degradation technologies

In recent years, the emergence of TPD strategies via the lysosomal pathway has been witnessed, including AUTAC, LYTAC, ATTEC, CMA-based degraders. These developments are driven by extensive research into the endosome-lysosome and autophagosome-lysosome pathways. Unlike proteasome-based TPD, which targets specific intracellular proteins, lysosome-based TPD can eliminate protein aggregates, damaged organelles, membranes, and extracellular proteins.

##### Autophagy-targeting chimeras (AUTACs)

AUTACs have been demonstrated to successfully degrade proteins and fragmented mitochondria by lysosome pathway.^242–244^ Inspired by the innate autophagic clearance of group A streptococcus, Arimoto’s group uncovered the role of 8-nitroguanosine 3′,5′-cyclic monophosphate (8-nitro-cGMP), which recruits autophagosomes mediated by Lys63-linked polyubiquitination.^245^ Ubiquitinated substrates are recognized by the autophagy receptor SQSTM1/p62 and interact with LC3, leading to their degradation in autophagosomes through selective autophagy. Cysteine residues can be modified by *S*-guanylation with 8-nitro-cGMP. Thus, endogenous cGMP modification (*S*-guanylation) could be a tag that targets proteins and mitochondria for autophagy. Given the crucial role of 8-nitro-cGMP, AUTACs could be designed to degrade fragmented mitochondria as well as proteins. The composition of the AUTAC molecule includes a guanine derivative-based degradation tag, a linker, and a warhead for binding to POI or organelle specificity. Therefore, AUTAC molecule initiates K63-linked polyubiquitination, leading to lysosome degradation. In 2019, Arimoto et al. first developed AUTAC1-4 and validated the concept of AUTAC; endogenous cGMP modification (S-guanylation) was utilized as tag for autophagy. However, the effect of AUTACs was limited because protein kinase G (PKG) could be activated by cGMP substructure and the poor cell membrane permeability. Arimoto et al. developed second-generation AUTACs in 2023 by optimizing guanine as degradation tag, the length of linker and L-Cys as connector.^246^ These optimizations significantly improved second-generation AUTACs degradation efficiency.

##### Lysosome targeting chimeras (LYTACs)

LYTAC is another promising technology that delivers extracellular proteins and membrane-bound proteins through the endosome-lysosome pathway for degradation. Lysosome-targeting receptors (LTR) facilitate the transport of proteins to lysosomes. In 2020, Bertozzi’s group pioneered the development and synthesis of the first LYTACs, innovative chimeric molecules that can bind simultaneously to a cell-surface LTR and an extracellular protein. This dual binding capability facilitates the internalization and subsequent lysosomal degradation of the targeted protein. Structurally, a LYTAC is composed of one end anchored to an LTR on the cell surface and the other end bound to the protein of interest, with both ends connected via a chemical linker. The formation of this trimeric LTR/LYTAC/protein of POI complex designates it for degradation by lysosomal protease enzymes.^136^ Soon after that, Bertozzi’s group and Tang et al. designed series of GalNAc-LYTACs. This liver-specific LYTAC further increases the variety of lysosomal targeting receptors, suggesting the potential for creating more cell-type-specific LYTACs. In 2023, Bertozzi et al. revealed some mechanisms of mediating the LYTAC degraders. The activation of the retromer complex, which recycles LYTAC–CI-M6PR complexes, could competitively inhibit LYTAC activity. The process of neddylation of cullin 3 (CUL3) is critical for delivering LYTAC to lysosomes and is considered as a biomarker for LYTAC degradation efficiency. LYTAC degradation could also be counteracted by mannose 6–phosphate (M6P) occupying CI-M6PR.^247^ These results could help to develop next-generation LYTACs.

##### Autophagosome-tethering compound (ATTEC)

ATTECs are a novel class of therapeutic molecules designed to harness the cell’s autophagy pathway for the targeted degradation of specific proteins. Autophagy is a critical cellular process that involves the degradation and recycling of cellular components through lysosomes. ATTECs specifically promote the binding of designated proteins to autophagosomes, the vesicles that capture cellular material destined for degradation. This targeted approach enables selective degradation of proteins that are associated with various diseases, particularly those where protein accumulation is pathogenic, such as in neurodegenerative diseases. By directing troublesome proteins directly to autophagosomes, ATTECs circumvent some usual cellular pathways, potentially reducing side effects and enhancing the specificity and efficiency of the autophagy system.^248^ To treat the incurable neurodegenerative disorder Huntington’s disease, Lu et al. put forward the ATTEC concept in 2019.^249^ Compared with other lysosomal targeting degradation technologies, ATTECs have small molecular weight and could degrade lipid, DNA/RNA and other substances more than protein.^250^ These advantages demonstrate that ATTECs could have a wide range of application in treatment.

##### CMA

CMA is a lysosomal degradation pathway that maintains proteostasis. CMA specifically degrades cytoplasmic proteins containing KFERQ-like motifs selected by chaperones (heat-shock cognate protein 70 recognition, HSC70), directly translocating across the lysosome membrane via lysosome-associated membrane protein type 2A (LAMP2) for degradation.^251^ Utilizing CMA mechanism, CMA-based degraders can be designed rationally for reducing endogenous proteins, which are difficult for small molecules to reach, such as abnormal proteins related to neurodegenerative diseases.^252^ CMA-based degraders are composed of three functional domains: a cell membrane penetration domain (CMPD), a target protein binding domain, and a CMA-targeting motif (CTM).

The above four technologies have been applied to TPD. In 2014, Wang et al. first verified the concept of CMA-based strategy and successfully degraded the target protein.^253^ The well-understood about CMA helps in designing some effective CMA-based degraders, however, the stability and transmembrane ability of degraders are still factors that need to be considered.^137,254^

Though it has greatly expanded the TPD application, lysosome-based TPD is still in the proof-of-concept stage. It’s a long way for lysosome-based TPD to clinical research, and it deserves in-depth study. We have summarized their characteristics and compiled them in Table 3.

**Table 3:** The characteristics of different lysosome-based degraders

Feature	AUTAC	LYTAC	ATTEC	CMA-based degrader
Mechanism	Utilize autophagy pathways to degrade target proteins or organelles	Degrade cell surface proteins via lysosomal pathways	Guide proteins to autophagosomes for degradation	Direct lysosomal degradation via chaperone proteins
Target types	Intracellular proteins and organelles	Cell surface proteins	Intracellular proteins	Intracellular proteins with KFERQ sequence
Poven target	Metalloprotease, dysfunctional mitochondria	Extracellular protein, transmembrane glycoprotein, immune checkpoint, kinase	PolyQ expansion proteins, kinase, lipid droplets	Proteins, α-synuclein, kinase
Advantage	Remove targeted cytosolic proteins or mitochondria in xenophagy	Degrade the extracellular secreted proteins and plasma membrane-associated proteins; can be liver-specific	Allele selective for a specific protein	Possible solution for the treatment of diseases caused by misfolded proteins
Limitation	The mechanism of K63 polyubiquitination induced by S-guanylation is still unknown	Cannot be applied to intracellular targets	The binding site in LC3 is not yet known	The delivery and stability of degrader peptides need to be resolved

## Application of TPD in human diseases

**Fig. 4:**
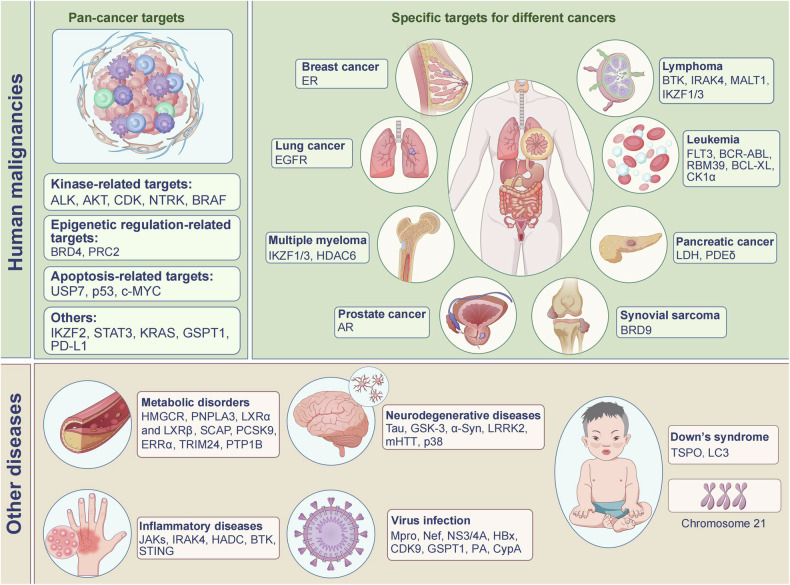
Targets of TPD in human diseases. This diagram illustrates key protein targets for various diseases including malignancies and other conditions such as metabolic disorders, neurodegenerative diseases, inflammatory diseases, viral infections, and Down’s syndrome. The diagram encompasses broad targets for cancers as well as specific targets for individual diseases

**Fig. 5:**
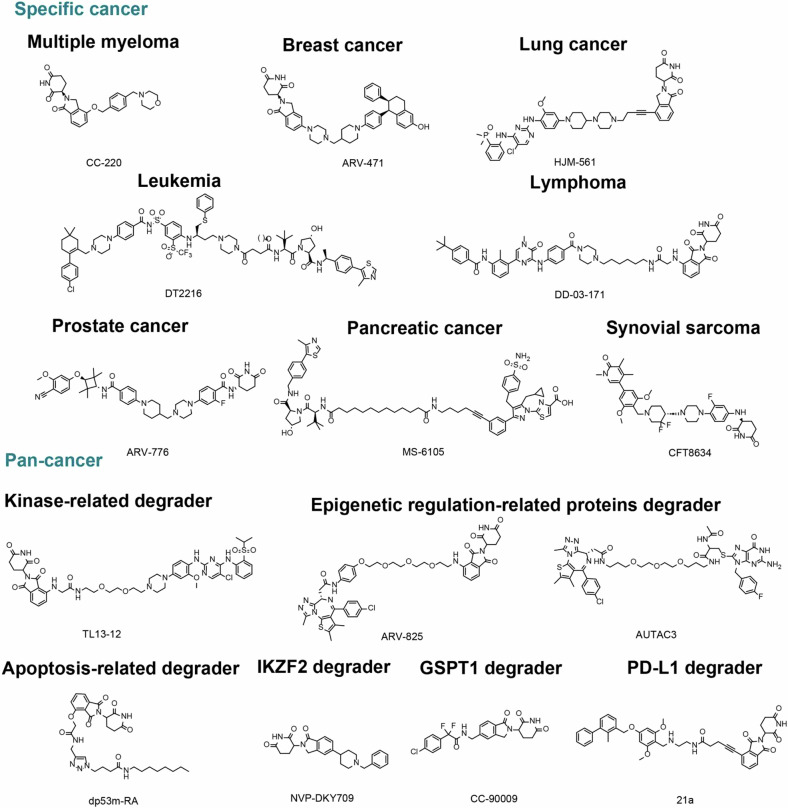
Structural representations of TPD compounds for treating various malignancies. CC-220 functions as an IKZF1/3 degrader for MM; ARV-471 degrades ER in breast cancer; HJM-561 targets EGFR for lung cancer; DT2216 targets BCL-XL in leukemia; DD-03-171 acts on BTK for lymphoma; ARV-776 is an AR degrader for prostate cancer; MS-6105 degrades lactate dehydrogenase in pancreatic cancer; CFT8634 targets BRD9 in synovial sarcoma. For pan-cancer applications, TL13-12 (ALK degrader) addresses kinase-related malignancies; ARV-825 and AUTAC3 (BRD4 degraders) focus on epigenetic regulation; dp53m-RA degrades p53, related to apoptosis; NVP-DKY709 targets IKZF2; CC-90009 is a GSPT1 degrader; 21a degrades PD-L1

**Fig. 6:**
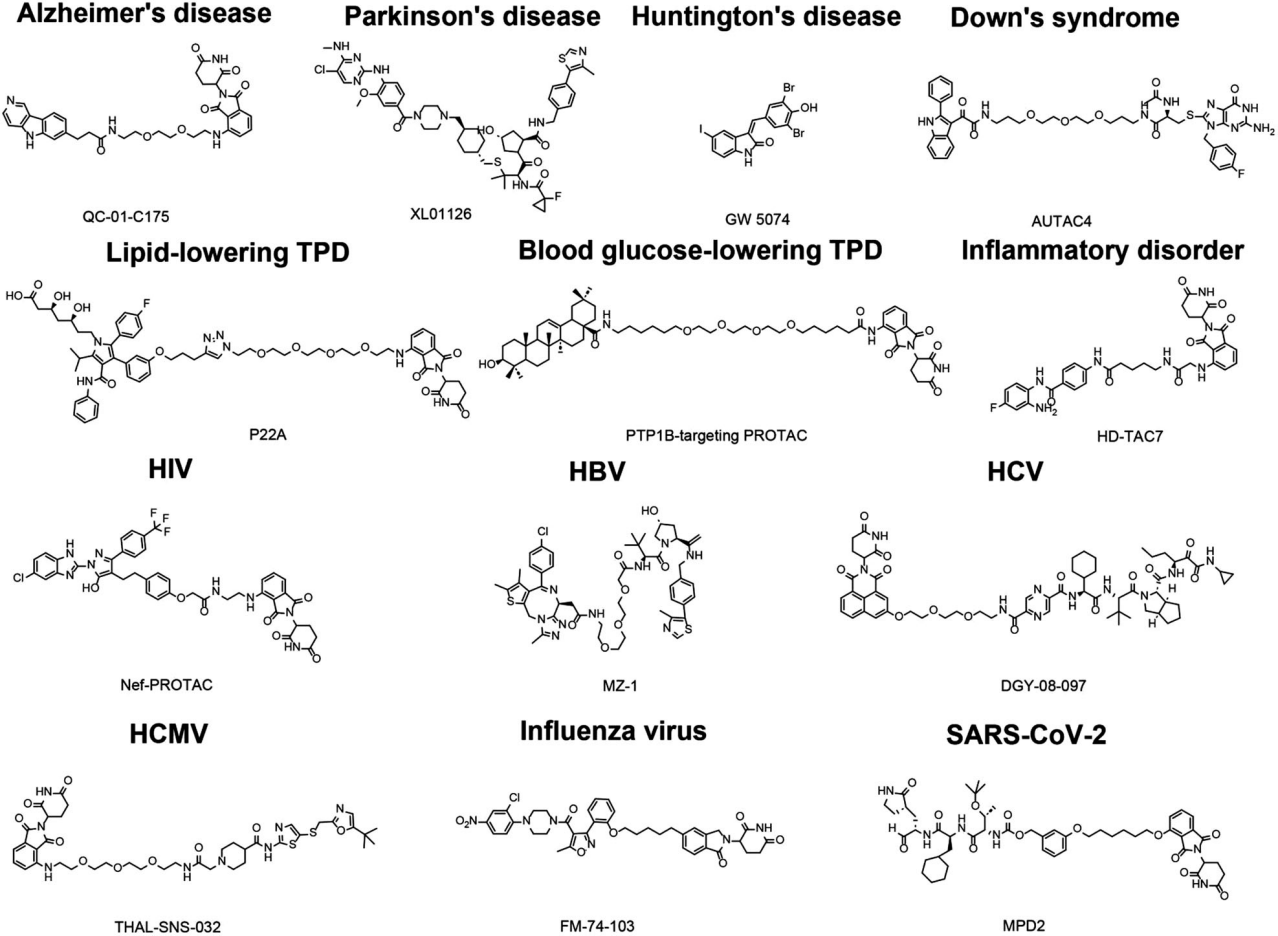
Structural representations of TPD compounds for the treatment of non-oncological diseases. Neurodegenerative Diseases: QC-01-C175, a tau degrader for Alzheimer’s disease; XL01126, a LRRK2 degrader for Parkinson’s disease; GW 5074, an mHTT degrader for Huntington’s disease. Down’s Syndrome: AUTAC4, which degrades dysfunctional mitochondria via the lysosome pathway. Metabolic Disorders: P22A, a HMG-CoA reductase degrader for lipid-lowering; PTP1B-targeting PROTAC for blood glucose reduction; HD-TAC7, an HDAC degrader for inflammatory disorders. Viral Infections: Nef-PROTAC, a HIV Nef degrader; MZ-1, a BRD4 degrader for HBV; DGY-08-097, an NS3/4 degrader for HCV; THAL-SNS-032, a CDK9 degrader for HCMV; FM-74-103, a GSPT1 degrader for influenza; MPD2, an Mpro degrader for SARS-CoV-2

TPD has emerged as a revolutionary strategy in the management of human diseases, driven by substantial improvements in structural optimization and cutting-edge screening technologies. These advancements have significantly enhanced the specificity and efficacy of TPD agents, making them a focal point in the ongoing fight against various malignancies and other complex disorders. Currently, numerous TPD agents are undergoing clinical trials, which demonstrate their potential as a potent new class of therapeutics. We have summarized the typical TPD targets in human diseases (Fig. 4), with agents currently in clinical trials listed in Table 4 and specific results detailed in Table 5. The structures of some key compounds for malignant diseases are depicted in Fig. 5, while the structures for other diseases are listed in Fig. 6.

**Table 4:** TPD in clinical trials for diseases

Target	Modality	Drug	Indication	Administration	Status	Trial number
IKZF1/3	MG	ICP-490	MM and lymphoma	Oral	Phase I/II	NCT05719701
		CC-122	Liquid and ST	Oral	Phase I/II	NCT01421524, NCT03834623, NCT02509039, NCT02323906, NCT02417285, NCT02859324, NCT02406742, NCT03283202, NCT05688475, NCT03310619, NCT02031419
		CC-99282	Lymphoma	Oral	Phase I/II	NCT06425302, NCT05788081, NCT06035497, NCT04434196, NCT06108232, NCT05169515, NCT06209619, NCT04884035, NCT03310619, NCT03930953, NCT06271057, NCT06356129, NCT05283720
		CC-92480	MM	Oral	Phase III	NCT02343042, NCT06121843, NCT06050512, NCT06163898, NCT05519085, NCT05552976, NCT05981209, NCT03989414, NCT06048250, NCT05372354, NCT03374085, NCT06627751
		CC-220	MM and lymphoma, SLE	Oral	Phase III	NCT02185040, NCT03161483, NCT04464798, NCT02773030, NCT05169515, NCT05199311, NCT04884035, NCT03310619, NCT04855136, NCT05560399, NCT04975997, NCT04392037, NCT06215118, NCT05827016, NCT04564703, NCT06107738, NCT05272826, NCT06121843, NCT05434689, NCT06232044, NCT06348108, NCT06465316, NCT06179888, NCT04776395, NCT05392946, NCT06518551, NCT05083520
		CFT7455	MM and lymphoma	Oral	Phase I/II	NCT04756726
RBM39	MG	E7820	Lipid and ST	Oral	Phase II	NCT01773421, NCT05024994, NCT00078637, NCT00309179
BCL-XL	PROTAC	DT2216	Liquid and ST	Intravenous	Phase I	NCT04886622, NCT06620302
BTK	PROTAC	AC676	R/R B-cell malignancies	Oral	Phase I	NCT05780034
		BGB-16673	R/R B-cell malignancies	Oral	Phase I	NCT05294731, NCT05006716, NCT06634589
		NX-5948	R/R B-cell malignancies	Oral	Phase I	NCT05131022
		ABBV-101	R/R B-cell malignancies	Oral	Phase I	NCT05753501
		HSK-29116	R/R B-cell malignancies	Oral	Phase I	NCT04861779
BTK, IKZF1/3	PROTAC	NX-2127	R/R B-cell malignancies	Oral	Phase I	NCT04830137
AR	PROTAC	AC176	mCRPC	Oral	Phase I	NCT05241613, NCT05673109
		ARV-110	mCRPC	Oral	Phase I/II	NCT05177042, NCT03888612
		ARV-766	mCRPC	Oral	Phase I/II	NCT05067140
		CC-94676	mCRPC	Oral	Phase I	NCT04428788, NCT06417229
		HP518	mCRPC	Oral	Phase I	NCT05252364, NCT06155084
ER	PROTAC	SIM0270	ER + /HER2- breast cancer	Oral	Phase I	NCT05293964
		ARV-471	ER + /HER2- breast cancer	Oral	Phase III	NCT05573555, NCT05548127, NCT05501769, NCT04072952, NCT05732428, NCT05463952, NCT06125522, NCT05549505, NCT05909397, NCT05654623, NCT06206837, NCT01042379
		AC682	ER + /HER2- breast cancer	Oral	Phase I	NCT05489679, NCT05080842
EGFR	PROTAC	HSK-40118	EGFR mutation NSCLC	Oral	Phase I	NCT06050980, NCT06536400
BRD9	PROTAC	FHD-609	Advanced synovial sarcoma	Intravenous	Phase I	NCT04965753
		CFT8634	Advanced synovial sarcoma	Oral	Phase I/II	NCT05355753
IKZF2	MG	DKY709	ST	Oral	Phase I	NCT03891953
GSPT1	MG	CC-90009	AML and MDS	Intravenous	Phase I	NCT02848001, NCT04336982
		MRT-2359	MM and lung cancer	Oral	Phase I	NCT05546268
GSPT1, IKZF1/3 and CK1α	MG	BTX-1188	Lipid and ST	Oral	Phase I	NCT05144334
KRAS G12D	PROTAC	ASP-3082	ST	Intravenous	Phase I	NCT05382559
STAT3	PROTAC	KT-333	Liquid and ST	Intravenous	Phase I	NCT05225584
NTRK	PROTAC	CG001419	ST	Oral	Phase I/II	CTR20222742
BRAF (V600E)	PROTAC	CFT1946	ST	Oral	Phase I/II	NCT05668585
BRD4	PROTAC	RNK05047	Advanced ST including DLBCL	Intravenous	Phase I/II	NCT05487170
MDM2, p53	PROTAC	KT-253	Liquid and ST	Intravenous	Phase I	NCT05775406
IRAK4	PROTAC	KT-413	DLBCL (MYD88-mutant)	Intravenous	Phase I	NCT05233033

*AR* androgen receptor, *ER* estrogen receptors, *BTK* Bruton’s tyrosine kinase, *IKZF1/3* IKAROS family zinc finger 1/3, *BRD* BET bromodomain protein, *IRAK4* interleukin-1 receptor-associated kinase 4, *MDM2* mouse double minute 2 homolog, *KRAS* Kirsten rat sarcoma viral oncogene homolog, *EGFR* epidermal growth factor receptor, *NTRK* neurotrophic receptor tyrosine kinase, *BRAF* B-Raf proto-oncogene, serine/threonine kinase, *IKZF* IKAROS family zinc finger protein, *GSPT1* G1 to S phase transition 1, *CK1α* casein kinase 1 alpha, *RBM39* RNA binding motif protein 39, *mCRPC* metastatic castration-resistant prostate cancer, *R/R* relapsed/refractory, *NHL* non-Hodgkin lymphoma*, ST* solid tumor, *DLBCL* diffuse large cell lymphoma, *NSCLC* non-small cell lung cancer, *MM* multiple myeloma, *AML* acute myeloid leukemia, *SLE* systemic lupus erythematosus, *MDS* myelodysplastic syndromes

**Table 5:** The clinical efficacy and safety of TPD in diseases

Drug	Modality	Target	Indication	Combination	Status	Patient number	Median follow-up (months)	Efficacy	≥G3 AEs	NCT number	Reference
ARV-110	PROTAC	AR	mCRPC	\	Phase I/II	Phase I: 67 Phase II: 106	≥6	AR T878A/S and/or H875Y cohort (*n* = 7) 2PR	Nausea (1%) Fatigue (1%) Vomiting (1%) Diarrhea (2%)	NCT03888612	^363^
ARV-471	PROTAC	ER	Advanced ER + /HER2- breast cancer	\	Phase I/II	Phase I: 60 Phase II:71	≥18	Phase I CBR 40%, 3PR Phase II 200 mg cohort (*n* = 35) CBR 37.1% with ESR1 mutation cohort CBR 47.4%, 3PR 500 mg cohort (*n* = 36) CBR 38.9% with ESR1 mutation cohort CBR 57.5%, 1PR	Phase I Headache (1%), Increased amylase and lipase (1%) QT prolongation (1%) Venous embolism (1%) Phase II 200 mg cohort (n = 35) QT prolonged (1%) Thrombocytopenia (1%) Hyperbilirubinemia (1%) 500 mg cohort (*n* = 36) Fatigue (1%) Decreased appetite (1%) Neutropenia (1%)	NCT04072952	^352,708^
BGB-16673	PROTAC	BTK	R/R B-cell malignancies	\	Phase I	26	3.5	All cohorts (*n* = 18) ORR 67% 1CR	Neutropenia (15.4%) Lipase increased (3.8%)	NCT05006716	^322^
NX-5948	PROTAC	BTK	R/R B-cell malignancies	\	Phase Ia/b	Phase Ia: 14	\	CLL evaluable cohort (*n* = 3) 1PR, 2 SD	No ≥G3 TRAEs	NCT05131022	^323^
NX-2127	PROTAC	BTK, IKZF1/3	R/R B-cell malignancies	\	Phase Ia/b	47	9.5	NHL evaluable cohort 2CR, 1PR	Neutropenia (38.3%) Hypertension (14.9%) Anemia (12.8%)	NCT04830137	^324^
KT-333	PROTAC	STAST3	R/R lymphoma, LGL-L, and ST	\	Phase Ia/b	21	\	Dmax DL1 (*n* = 4): 69.9% DL2 (*n* = 3): 73.5%, 1PR DL3 (*n* = 3): 79.9% DL4 (*n* = 4): 86.6%	No ≥G3 TEAs	NCT05225584	^505^
KT-474	PROTAC	IRAK4	HS and AD	\	Phase Ia/b	21	2w	HS evaluable cohort (*n* = 12) HiSCR50: 42–50% Nodule count reduction: 46.1–50.7% AD evaluable cohort (*n* = 7) Peak Pruritus response rate: 71% Peak pruritus declined rate: 62.9%	\	NCT04772885	^593^
CC-122	MG	IKZF1/3	Advanced ST, NHL, and MM	\	Phase I	34	\	NHL evaluable cohort (*n* = 5) ORR 60%, CR 20%, PR 40%	Neutropenia (27%) Pneumonia (6%)	NCT01421524	^337^
			NHL	\	Phase I	97	\	R/R DLBCL (*n* = 84) ORR 29%, CR 11%	Neutropenia (51%) Infections (24%) Anemia (12%) Febrile neutropenia (10%)	NCT01421524	^336^
			R/R DLBCL, FL	CC-223, CC-292 and/or rituximab	Phase Ib	106	\	^a^Arm A (*n* = 31) ORR 29.0%, CR 12.1% Arm B (*n* = 27) ORR 25.9%, CR 11.1% Arm C (*n* = 14) ORR 0% Arm D (*n* = 30) ORR 23.3%, CR 7%	Arm A (*n* = 31) Neutropenia (45.2%) Anemia (12.9%) Thrombocytopenia (16.1%) Febrile neutropenia (9.7%) Diarrhea (19.4%) Arm B (*n* = 27) Neutropenia (44.4%) Anemia (18.5%) Thrombocytopenia (25.9%) Febrile neutropenia (18.5%) Arm C (*n* = 14) Neutropenia (14.3%) Anemia (14.3%) Thrombocytopenia (28.6%) Hypotension (28.6%) Arm D (*n* = 30) Neutropenia (36.7%) Thrombocytopenia (13.3%) Febrile neutropenia (6.7%)	NCT02031419	^340^
			R/R NHL	Obinutuzumab	Phase Ib	73	8.4	All cohorts (*n* = 73) ORR 68%, CR 34% DLBCL (*n* = 19) ORR 47%, CR 11% FL (*n* = 53) ORR 76%, CR 43%	Neutropenia (56%) Thrombocytopenia (23%)	NCT02417285	^339^
			NHL, ST	\	Phase I	15	\	NHL (*n* = 13) ORR 54%, CR 31%	Decreased neutrophil count (33%) Lymphocyte count (20%)	NCT02509039	^338^
CC-220	MG	IKZF1/3	R/R MM	DEX	Phase I/II	Phase I: 90 Phase II: 107	Phase I: 5.8 Phase II: 7.7	Phase I ORR 32%, CR 1%, PR 31% Phase II ORR 26%, CR 1%, PR 26%	Neutropenia (45%) Anemia (28%) Infections (27%) Thrombocytopenia (22%)	NCT02773030	^267^
			R/R lymphoma	Rituximab or obinutuzumab	Phase I/II	46	\	All cohorts (*n* = 38) ORR 55%, CR 32% ^b^Cohorts B ORR 71%, CR 29% Cohorts C ORR 69%, CR 39%	Neutropenia (49%) Anemia (15%) Thrombocytopenia (13%)	NCT04464798	^266^
			Newly diagnosed transplant-eligible MM	Carfilzomib and DEX	Phase I/II	6	3	1 CR + sCR and MRD-negative	Neutropenia (16.7%)	NCT05199311	^268^
CC-92480	MG	IKZF1/3	R/R MM	DEX	Phase I/II	Phase I: 77 Phase II: 101	7.5	Phase I (*n* = 77) ORR 25%, CR 1%, PR 24% Phase II (*n* = 101) ORR 41%, CR 5%, PR 36%	Phase I (*n* = 77): Neutropenia (71%) Anemia (38%) Thrombocytopenia (24%) Febrile (9%) Infections and infestations (40%) Phase II (*n* = 101): Neutropenia (76%) Anemia (36%) Thrombocytopenia (28%) Febrile (15%) Infections and infestations (35%)	NCT03374085	^271^
			R/R MM	BORT + DEX	Phase I/II	19	8	All cohorts (*n* = 19) ORR 73.7%	Neutropenia (36.8%), Thrombocytopenia (21.1%) Anemia (10.5%) Hyperglycemia (10.5%) Insomnia (10.5%)	NCT03989414	^272^
CC-99282	MG	IKZF1/3	R/R NHL	\	Phase I	35	\	All cohorts (*n* = 25) ORR 40%, CR 12%, PR 28%	Neutropenia (54%), Thrombocytopenia (9%) Febrile neutropenia (6%)	NCT03930953	^332^
CFT7455	MG	IKZF1/3	R/R MM, NHL	\	Phase I/II	5	\	Sustained degradation of IKZF3 (around 100%) A reduction in serum free light chains (up to 72%)	Neutropenia (60%)	NCT04756726	^275^
CC-90009	MG	GSPT1	R/R AML	\	Phase I	45	\	1CR, 1CRi,1MLFS	Hypocalcemia (22%) Hypotension (13%)	NCT02848001	^488,489^
E7820	MG	RBM39	R/R splicing factor-mutant AML, MDS, or CMML	\	Phase II	12	13.1	1CRi	Anemia (16.7%) Neutropenia (16.7%) Hematoma (16.7%) Lung infection (16.7%) Flatulence (16.7%) Respiratory failure (16.7%)	NCT05024994	^131^

*AR* androgen receptor, *ER* estrogen receptor, *BTK* Bruton’s tyrosine kinase, *STAT3* signal transducer and activator of transcription 3, *IKZF1/3* IKAROS family zinc finger 1/3, *GSPT1* G1 to S phase transition 1, *RBM39* RNA binding motif protein 39, *mCRPC* metastatic castration-resistant prostate cancer, *R/R* relapsed/refractory, *LGL-L* large granular lymphocytic leukemia, *ST* solid tumors, *NHL* non-Hodgkin lymphoma, *MM* multiple myeloma, *DLBCL* diffuse large b-cell lymphoma, *FL* follicular lymphoma, *HS* hidradenitis suppurativa, *AD* atopic dermatitis, *AML* acute myeloid leukemia, *MDS* myelodysplastic syndromes, *CMML* chronic myelomonocytic leukemia, *BORT* bortezomib, *DEX* dexamethasone, *PR* partial response, *CBR* clinical benefit rate, *ORR* overall response rate, *CR* complete response, *SD* stable disease, *Dmax* maximum degradation, *DL* dose levels, *HiSCR50* HS clinical response 50%, *sCR* stringent complete response, *MRD* minimal residual disease, *CRi* complete remission with incomplete hematologic recovery, *MLFS* morphologic leukemia-free state, *G3* grade 3, *AEs* adverse effect, *TRAEs* treatment-related adverse events

^a^Arm A, avadomide + CC-223 ± rituximab; Arm B, avadomide + CC-292 ± rituximab; Arm C, CC-292 + CC-223; Arm D, avadomide + rituximab

^b^Cohorts B, CC-220 and rituximab; Cohorts C, CC-220 and obinutuzum

### TPD in hematologic malignancies

Hematological malignancies, including lymphoma, leukemia, and myeloma, are a group of malignancies originating from the bone marrow or lymphatic system. The treatment of these diseases is complex and often requires targeting specific molecular markers. Against this backdrop, TPD represents an unprecedented therapeutic approach for hematological malignancies, demonstrating great potential by precisely degrading pathogenic proteins.

#### Multiple myeloma (MM)

MM is a blood cancer characterized by aberrant cells accumulating in the bone marrow, which suppresses the production of healthy blood cells and leads to complications such as bone loss and kidney damage. Despite available treatments like chemotherapy and targeted therapies, MM remains highly recurrent and hard to cure. TPD offers a novel therapeutic approach for MM, particularly through the degradation of key proteins.

##### Ikaros family zinc finger proteins 1/3 (IKZF1/3) degraders

IKZF1/3 are critical transcription factors within the Ikaros family, known for their zinc finger domains. They play vital roles in B-cell development and the regulation of immune responses. In addition, their degradation has been found to enhance IL-2 expression and the proliferation of NK and T cells, thereby offering an effective mechanism for modulating immune functions.^255–259^ IKZF1 and IKZF3 degraders, such as thalidomide and lenalidomide, have been recognized for their therapeutic efficacy in MM.^260^ The structural elucidation of the DDB1-CRBN-lenalidomide complex^68^ has propelled forward our molecular understanding and the development of effective TPDs. It exhibits their therapeutic potential that several promising cereblon E3 ligase modulators (CELMoDs) are undergoing clinical trials now.

CC-220 enhances the degradation of IKZF1 and IKZF3,^261^ showing significant anti-proliferative activity in various diseases, particularly in systemic lupus erythematosus and R/R MM.^262–264^ Preclinical studies indicate that CC-220 is more effective than bortezomib and pomalidomide, especially in combination with daratumumab, where it shows synergistic effects against resistant MM cells.^265^ In clinical trials, CC-220 has demonstrated promising efficacy and safety profiles. Early phase trials established a maximum tolerated dose of 3.0 mg daily and showed an overall response rate (ORR) of 55% in lymphoma patients, with enhanced responses when combined with CD20 monoclonal antibodies such as rituximab or obinutuzumab. Specifically, the ORR for the combination with rituximab was 71%, and it was 69% for the combination with obinutuzumab.^266^ Further studies in R/R MM patients evaluated the combination of CC-220 with dexamethasone. The ORR was 32% in the dose-escalation cohort and 26% in the dose-expansion cohort, confirming the efficacy of CC-220 in multi-drug regimens.^267^ Ongoing Phase III studies are exploring CC-220’s efficacy in combination with daratumumab and dexamethasone for R/R MM and its role in maintenance therapy with lenalidomide post-allogeneic stem cell transplantation (NCT04975997, NCT05827016). These trials aim to further validate CC-220’s role in enhancing treatment outcomes across various complex treatment landscapes. Moreover, the CC-220 regimen, when combined with carfilzomib and dexamethasone, demonstrated promising results in a cohort of newly diagnosed, transplant-eligible MM patients. A significant CR was achieved by one evaluated patient, suggesting that deep remissions are possible with this regimen.^268^

CC-92480, a novel CELMoD, has demonstrated superior binding affinity and degradation efficacy, particularly in lenalidomide-resistant MM cell lines.^269^ In combination with bortezomib and dexamethasone, it greatly enhances T and NK cell activation, significantly improving tumor cell eradication.^270^ In clinical trials, CC-92480 combined with dexamethasone has shown promising efficacy and tolerability in triple-class-refractory MM. Phase I results indicated an ORR of 25%, while Phase II revealed an ORR of 40.6% among 101 evaluable patients. The drug was particularly effective in patients benefiting from BCMA-targeted therapy.^271^ Another Phase Ib study, which combined CC-92480 with bortezomib and dexamethasone in R/R MM achieved an ORR of 73.7%. This highlights its potential for high efficacy and a manageable safety profile, even in challenging cases.^272^ Ongoing Phase III studies are further assessing CC-92480’s effectiveness in combinations with carfilzomib and dexamethasone or bortezomib, aiming to establish robust treatment regimens for diverse MM scenarios. In addition, CC-92480 is also effective in overcoming IMiD resistance in T-cell lymphomas by degrading both IKZF1 and ZFP91, which are crucial for T cell lymphomas survival.^273^ These studies validate the enhancement of CC-92480 role in MM and potentially other hematologic malignancies treatment.

CFT7455, a novel therapeutic, shows an 800 to 1600-fold higher binding affinity to CRBN compared to pomalidomide. In preclinical studies, it exhibited strong anti-proliferative effects in MM cell lines, including those resistant to IMiDs. Notably, in an RPMI-8226 MM mouse xenograft model, CFT7455 profoundly and persistently degraded IKZF3, and its combination with Dexamethasone significantly enhanced anti-tumor efficacy.^274^ Similarly, in non-Hodgkin's lymphoma (NHL) models unresponsive to pomalidomide, CFT7455 demonstrated significant degradation capabilities.^275^ Ongoing Phase I/II trials for R/R NHL and MM have shown promising early results, with near-complete, sustained IKZF3 degradation and up to 72% reduction in serum free light chains. Despite these benefits, severe neutropenia (grade 4) occurred in three out of five patients, prompting investigations into alternative dosing regimens to improve safety and therapeutic indices.^275^ Furthermore, CFT7455 enhanced T-cell activation, cytokine secretion, and ADCC/TDCC activities, suggesting beneficial interactions with mAbs and bispecific T-cell engagers such as daratumumab and teclistamab.^276^ This synergy could potentially improve therapeutic outcomes in MM, supported by ongoing clinical evaluations.

ICP-490 is a novel CELMoD with high potency and oral bioavailability. It selectively degrades IKZF1 and IKZF3 at sub-nanomolar concentrations, exhibiting significant efficacy against various MM and DLBCL cell lines and xenograft models, even against lenalidomide-resistant cells. Notably, ICP-490 exhibits no obvious cytotoxicity in normal cells, SD rats and cynomolgus monkey.^277^ Currently, ICP-490 is being evaluated in a Phase I/II clinical trial for R/R MM.

CELMoDs surpass traditional IMiDs by featuring enhanced binding affinities and efficient protein degradation mechanisms, which help to overcome drug resistance and minimize off-target effects. These properties could enhance patient safety and broaden the scope of treatable cancers, including solid tumors.

##### Histone deacetylases 6 (HDAC6) degraders

HDAC6, a histone-modifying enzyme, mainly regulates gene transcription. Inhibiting HDAC6 has demonstrated efficacy in the treatment of MM^278,279^ by disrupting pathways that lead to the accumulation of toxic protein aggregates,^280^ thereby inducing cancer cell death. However, the non-selectivity and potential for drug resistance associated with these inhibitors have driven the development of more targeted HDAC degraders.^281^

In 2018, the first HDAC6 degraders were developed by conjugating a pan-HDAC inhibitor with thalidomide analogs, leading to selective degradation of HDAC6 in MM.^282^ Various potent HDAC6 degraders were created by using the HDAC6-specific inhibitor nexturastat A, demonstrating promising anti-proliferation activity in MM cells.^283,284^ However, IKZF1/3 degradation was also observed in these studies. Considering this effect of thalidomide analogs, Yang et al. introduced a substituted phenyl ring to thalidomide to promote the selective degradation of HDAC6.^285^ Besides, VHL-based PROTACs also displayed selective degradation at nanomolar half-maximal degradation concentration (DC_50_) without significant cytotoxicity.^285^ Hansen’s group provided an alternative synthetic way.^286^ They employed innovative solid-phase synthesis approach to create both hydroxamic acid-based and non-hydroxamic acid-based PROTACs with potent degradation but suboptimal cell cytotoxicity in MM.^287,288^

#### Leukemia

Leukemia is a malignant tumor originating from the hematopoietic system, characterized by the abnormal proliferation of immature white blood cells in the bone marrow and other blood-forming organs. These abnormal cells not only impair the production of normal blood cells but also invade other organs, which bring about multi-system dysfunction. TPD, as a new strategy, has shown significant therapeutic potential in leukemia.

##### Fms-like tyrosine kinase 3 (FLT3) degraders

FLT3, a receptor tyrosine kinase predominantly expressed in hematopoietic stem cells, is critical in mediating cell growth and survival through pathways including PI3K/AKT and MAPK. Mutations in FLT3-ITD, found in nearly 30% of AML patients, exacerbate disease progression and cell differentiation. Although several FLT3-ITD SMIs are clinically approved, their efficacy is often curtailed by resistance.^289^ Crew et al. developed FLT3-targeting PROTACs, combining pomalidomide with quizartinib, which effectively degraded FLT3 in MOLM-14 cells harboring FLT3-ITD mutations in vivo, albeit with less inhibition of downstream signaling compared to quizartinib alone.^290^ In 2021, a novel FLT3 degrader based on dovitinib and CRBN ligand demonstrated enhanced anti-proliferative effects, complete blockade of downstream signaling at low concentrations, and efficacy against KIT proteins.^9^ In 2022, Chen et al. synthesized a series of FLT3-targeting PROTACs. Compound PF15 emerged as the most potent, effectively suppressing FLT3-ITD-positive cells proliferation with minimal off-target effects. PF15 also degraded ITD-D835V and ITD-F691L mutations and was validated by xenograft model.^291^ Concurrently, Soural et al. designed a novel dual FLT3/CDK9-targeting PROTAC, based on the purine inhibitor BPA311, showing significant selectivity and efficacy comparable to its parent inhibitor in AML cells with FLT3-ITD mutations, although direct comparisons with FLT3 or CDK9 degraders were not conducted.^292^

##### BCR-ABL degraders

The fusion oncoprotein BCR-ABL is a key driver of continuous cell proliferation in CML, activating downstream signaling pathways such as PI3K/AKT signal transducer and MAPK.^293,294^ Despite BCR-ABL inhibitors transforming life-threatening CML into a manageable chronic condition, issues like resistance and lifelong medication persist. PROTACs present a promising alternative by targeting specific protein degradation to overcome these challenges.

In 2016, Crew et al. developed a series of BCR-ABL or c-ABL degraders using different warheads linked to VHL or CRBN ligands, achieving effective degradation and proliferation inhibition in K562 cells at micromolar concentrations. However, initial VHL-based Imatinib- recruiting and Bosutinib-recruiting PROTACs showed no degradation capability.^295^ Following structural modifications, GMB-475, an imatinib-recruiting PROTAC with enhanced cell permeability and affinity, displayed stronger anti-proliferation activity than imatinib in Ba/F3 cells harboring BCR-ABL mutations like T315I or G250E at sub-micromolar levels while maintaining safety for healthy CD34+ cells.^296^

In 2019, Jiang et al. reported SIAIS178, a potent PROTAC that degraded several BCR-ABL mutations and induced significant tumor regression in mice at nanomolar concentrations, albeit with similar efficacy to parent SMIs.^10^ In addition, SNIPERs, particularly SNIPER(ABL)-2 reported by Naito’s group in 2016, have shown remarkable efficiency in degrading BCR-ABL at nanomolar concentrations and reducing downstream signaling phosphorylation.^297^ Furthermore, innovations such as nimbolide, a ligand for E3 ligase RNF114, have been incorporated into PROTACs, preferentially degrading BCR-ABL over c-ABL.^90^ Photo-switchable PROTACs have also been developed for controllable degradation.^298^ Subsequent studies proposed a series of potent degraders targeting allosteric sites or demonstrated sustained effects after drug removed,^299^ offering the prospect of drug withdrawal in CML patients.

##### RNA-binding motif protein 39 (RBM39) degraders

RBM39, an RNA-binding protein, plays a critical role in transcriptional co-regulation and selective RNA splicing. The disruption of RBM39 leads to abnormal splicing events and altered gene expression, impacting cell cycle progression and promoting tumor regression.^300^ E7820, targets RBM39 and its homologous protein RBM32 for degradation via the DCAF15 E3 ubiquitin ligase pathway, displaying cytotoxic effects across various cancer cell lines.^301^ Despite sharing myelosuppressive side effects similar to Indisulam, E7820 offers improved oral bioavailability.

A Phase II clinical trial involving 12 patients with R/R splicing factor-mutant cancers (7 AML, 5 MDS) assessed the efficacy of E7820. After a median follow-up of 13.1 months, only one patient achieved a transient marrow complete response (CR) without hematologic improvement, with an OS of 3.8 months. The observed efficacy in patients was less than expected, with less than 50% RBM39 degradation efficiency compared to over 90% in preclinical models, highlighting the challenges of translating in vitro results to clinical outcomes. This discrepancy may be due to differences in drug metabolism, distribution, or the complex tumor microenvironment in patients.^131^ Given these challenges, exploring combination therapies could provide a more effective treatment strategy.

##### B-cell lymphoma-extra large (BCL-XL) degraders

BCL-XL, a member of the pro-survival BCL-2 protein family, is frequently upregulated in tumors, disrupting the apoptotic balance and promoting tumorigenesis.^302^ While inhibitors targeting these proteins are used in cancer therapy, their clinical utility is limited by significant toxicity due to BCL-XL overexpression in platelets. BCL-XL-targeting PROTACs offer a promising solution by reducing platelet toxicity, thanks to the limited expression of VHL and CRBN in platelets.^303,304^

In 2019, Zheng et al. developed XZ424, a CRBN-recruiting BCL-XL degrader that achieved 85% degradation efficiency at 100 nM in MOLT-4 cells, without affecting platelets.^305^ Concurrently, Zhou’s group synthesized DT2216, a PROTAC based on the dual BCL-XL and BCL-2 inhibitor ABT263 and a VHL ligase ligand. This compound showed low platelet toxicity and significant pro-apoptotic effects in T-cell acute lymphoblastic leukemia (T-ALL).^303^ When combined with chemotherapy, DT2216 was effective in T-cell lymphomas and enhanced survival rates in T-ALL mouse models. Particularly notable was its combination with venetoclax, which substantially extended survival times beyond individual treatments.^306^ Further studies revealed that various drug-resistant T-ALL cell lines remained sensitive to DT2216, indicating its potential as an effective therapy for R/R T-ALL, especially in combination with other treatments to enhance efficacy.^307^ Currently, DT2216 has entered Phase I clinical trials.

Furthermore, in 2021, Zheng et al. developed PZ703b, a dual degrader of both BCL-XL and BCL-2, demonstrating superior potency over ABT263 and DT2216 by effectively degrading BCL-XL and inhibiting BCL-2.^308^ Computational modeling promoted the synthesis of dual-targeted PROTACs, of which 753b emerged as the most potent in the Kasumi-1 cell line with cytarabine resistance.^308^ In addition, PROTACs PZ18753b and WH2544 exhibited significant pro-apoptotic activities in venetoclax-resistant or BCL-2 mutant CLL cells.^309^ BCL-XL-targeting PROTACs have shown considerable promise in reducing platelet toxicity and enhancing anti-tumor efficacy, especially in R/R patients. Further clinical trials are essential to fully ascertain the safety and effectiveness.

##### Casein kinase 1 alpha (CK1α) degraders

CK1α, a serine/threonine protein kinase, is a viable target for AML therapy for promoting AML progression by inhibiting p53 pathways.^310^ Research by Woo et al. promoted the development of an IKZF2 degrader, which emerged as a dual degrader of IKZF2 and CK1α through unbiased proteomics and PRISM screening assays. These dual degraders halt AML cell proliferation and induce myeloid differentiation via CK1α-p53 and IKZF2-dependent mechanisms, with their effectiveness confirmed in both AML cell transplanted mice models and cells form patients.^311^ Subsequently, PROTACs were developed that co-degrade CK1α and CDK7/9, stabilizing p53 and suppressing MYC, MCL-1, and MDM2. This led to the induction of apoptosis in AML and curbed tumor growth in PDX models.^312^ Nishiguchi’s development of SJ 3149, a selective and potent CK1α degrader, has shown extensive anti-proliferative effects across numerous cancer cell lines,^313^ expanding the therapeutic scope of selective CK1α degraders in oncology.

#### Lymphoma

Lymphoma, a cancer of the lymphatic system, is broadly classified into Hodgkin's lymphoma and NHL. These tumors, formed by the abnormal proliferation of lymphocytes, often require targeted therapies to inhibit specific signaling pathways. TPD technology can precisely regulate key signaling pathways in lymphoma, offering a potent new strategy for treatment.

##### Bruton’s tyrosine kinase (BTK) degraders

BTK, a crucial non-receptor tyrosine kinase in hematopoietic cells, is integral to pathways such as the B-cell receptor and Toll-like receptor signaling.^314^ Dysregulated BTK expression is pivotal in B-cell malignancies and autoimmune diseases, making it a prime target for anticancer therapies. Despite the approval of several BTK inhibitors, challenges including resistance and off-target effects persist.^315^

BTK-targeting PROTACs, leveraging the CRBN-binding drug pomalidomide and BTK inhibitor ibrutinib, demonstrated promising efficacy in degrading both wild-type and ibrutinib-resistant BTK mutant (C481S/T/G/W/A) in HeLa and HBL-1 cells, potentially avoiding off-target events seen with ibrutinib.^316^ Substituting pomalidomide with lenalidomide, Rao et al. developed L18I, which demonstrated enhanced solubility, broader degradation of BTK mutants, and potent anti-proliferative effects both in vitro and in vivo. Notably, L18I combined with dasatinib showed increased efficacy in ibrutinib-resistant cells.^6^ In addition, the PROTAC-MG hybrid DD-03-171, targeting both BTK and the regulatory factors IKFZ1/3, significantly improved survival rate in mouse models of diffuse large B-cell lymphoma (DLBCL) and mantle cell lymphoma (MCL).^317^ Recent innovations have introduced photocaged PROTACs for BTK, which enable controlled release and targeted degradation.^318^ The oral bioavailability of BTK-targeting PROTACs has been enhanced through structural optimizations. Compounds such as UBX-382 and NRX-0492 demonstrated robust antitumor activities and sustained effects post-withdrawal.^319,320^ Currently, at least six BTK-targeting PROTACs under clinical trials, showed promising efficacy.^321^ Three preliminary clinical trials presented at 2023 ASH annual meeting assessed the safety and efficacy of BTK degraders in B-cell malignancies^312^ BGB-16673 with 67% ORR in relapsed/refractory (R/R) B-cell malignancies, was not terminated due to adverse effects.^322^ Another compound, NX-5948, showed excellent tolerability with no serious adverse effects,^323^ while NX-2127 was discontinued due to safety concerns despite achieving lasting CR in NHL patients.^324^ In addition, a recent study has discovered that in patients with CLL, NX-2127 achieved more than 80% degradation of BTK, including mutated forms of the BTK protein. These holds promise for addressing resistance issues associated with BTK inhibitors.^325^ Ongoing trials continue to shape the potential of BTK-targeting PROTACs in treating B-cell malignancies, highlighting the need for further research to optimize their efficacy and safety profiles.

##### Mucosa-associated lymphoid tissue lymphoma translocation protein 1 (MALT1) degraders

MALT1, a key protein and protease in immune response regulation, functions as a subunit of CBM complex, which includes BCL10 and caspase recruitment domain-containing protein 11 (CARD11). The CBM complex could activate NF-κB by cleaving specific substrates.^326^ Abnormal activations or mutations in MALT1 and CARD11 are linked to various cancers, particularly B-cell lymphomas. Melnick et al. developed a series of PROTACs targeting MALT1 that demonstrated selective killing effects in ABC-DLBCL, compared to germinal center B-Cell DLBCL (GCB-DLBCL), degrading over 50% of MALT1 protein and suppressing NF-κB activation.^327^ In addition, Wang et al. showed that MALT1 can contribute to ibrutinib resistance through bypassing BTK/CARD11 signaling.^2^ Dual knockdown of BTK and MALT1 significantly enhanced antitumor effects in ibrutinib-resistant MCL cell lines. This strategy holds promise for overcoming drug resistance in clinical treatment, offering more effective therapies.

##### Interleukin-1 receptor-associated kinase 4 (IRAK4) degraders

IRAK4 plays a crucial role in the immune response by integrating with MYD88 in signaling complexes for Toll-like receptors (TLRs) and interleukin-1 (IL-1) receptors. This integration triggers cascades that activate pathways, including NF-κB and PI3K-AKT-mTOR,^328,329^ which are involved in various inflammatory, autoimmune, and cancerous conditions. As such, IRAK4, a central component of MYD88-dependent signaling, is a promising target for therapeutic intervention.

In 2020, Dai et al. developed CRBN-based IRAK4 degraders and evaluated their effects in activated B-cell-like DLBCL (ABC DLBCL) by modulating immune-related pathways. These degraders effectively reduced IRAK4 levels at 1 μM, and inhibited the NF-κB signaling pathway. While IRAK4 degraders showed potential in pathway modulation, they did not significantly affect cell apoptosis or growth.^330^ The ongoing Phase Ia study of KT-413 is further assessing their efficacy in DLBCL patients with MYD88 mutations. This research underscores the potential of IRAK4-targeting PROTACs as new therapeutic options for malignancies and immune-related disorders.

##### IKZF1/3 degraders

Currently, the role of IKZF1/3 degraders, especially cellular modulator of immune recognition (CELMoD), is being explored beyond MM, particularly in B-cell lymphomas and some T-cell lymphomas.

CC-99282 has demonstrated outstanding efficacy in preclinical trials, outperforming CC-122, lenalidomide, and iberdomide in various DLBCL subtypes. It effectively induces rapid and sustained degradation of IKZF1 and IKZF3, and apoptosis in malignant cells.^331^ In addition, synergistic effects have been observed when CC-99282 is combined with anti-CD20 monoclonal antibodies.^331^ In a Phase I study, CC-99282’s safety and efficacy were evaluated in patients with R/R NHL. The treatment was generally manageable. 60% of patients experienced significant but manageable hematologic side effects, primarily neutropenia. Despite these challenges, CC-99282 achieved an overall response rate (ORR) of 40%. The responses lasted between 9 and 407 days. Pharmacokinetic data confirmed rapid absorption and an extended half-life, supporting its potential for sustained efficacy.^332^ Ongoing clinical trials are exploring the combination of CC-99282 with other established therapies for CLL and NHL, which could provide more options for treatment.

CC-122 (Avadomide) has demonstrated substantial anti-proliferative activity across various DLBCL subgroups, surpassing lenalidomide in efficacy.^333^ This CELMoD uniquely degrades IKZF1 and boosts interferon-stimulated genes, enhancing tumoricidal activities^333,334^ and modulating the immune response by increasing PD-L1 expression. Combining Avadomide with PD-1/PD-L1 blockade has effectively reinvigorated exhausted T cells and improved their tumor-killing capacity.^335^ CC-122 has undergone extensive trials, initially establishing a maximum tolerated dose of 3.0 mg daily. It showed a promising pharmacodynamic profile with an acceptable safety profile, achieving significant responses in various cancers, including NHL^336^ and brain cancer.^337^ Further studies in Japan confirmed its efficacy and safety in advanced solid tumors and NHL, with an ORR of 54% and CR of 31% among evaluated NHL patients.^338^These findings underscore the safety and efficacy of CC-122 as a monotherapy, prompting further studies into combination therapies. A Phase I trial combining CC-122 with ocrelizumab in NHL indicated an ORR of 68%, showing higher efficacy in R/R follicular lymphoma compared to DLBCL. This combination therapy demonstrated manageable safety profiles, emphasizing its potential benefits for R/R conditions.^339^ In addition, a Phase Ib study explored a multi-drug regimen combining CC-122 with the rapamycin kinase inhibitor CC-223, BTK inhibitor CC-292, and rituximab. This regimen showed enhanced tumor growth inhibition in a DLBCL xenograft model, although it raised concerns about increased toxicities.^340^ These findings underscored the efficacy of CC-122, both as a monotherapy and in combination therapies.

Ongoing and future clinical trials are broadening therapeutic strategies for lymphoma, exploring combinations with other drugs such as lisocabtagene maraleucel, a CD19-targeted CAR-T therapy (NCT03310619), and the R-CHOP chemoimmunotherapy regimen (NCT03283202). These combinations aim to significantly enhance treatment efficacy. In addition, the potential of CC-292 is being extended to solid tumors, with its effects studied in combination with nivolumab in patients with unresectable hepatocellular carcinoma (HCC) (NCT02859324) and advanced melanoma (NCT03834623).

### TPD in solid tumors

TPD, as a transformative approach in the treatment of solid tumors, could prove a precise method to eliminate key oncoproteins, overcome the limitations of traditional therapies and create more effective and less toxic treatment options. As research progresses, TPD is increasingly recognized for its ability to target previously ‘undruggable’ proteins, promising to revolutionize the management of various solid cancers.

#### Breast cancer

Breast cancer remains one of the most prevalent malignancies affecting women worldwide. Estrogen receptors are overexpressed in approximately 70-80% of breast cancer cases,^341,342^ making them a cornerstone of targeted treatment strategies. Despite the effectiveness of selective estrogen receptor modulators in estrogen receptor-positive breast cancer, resistance remains a challenge.^343^ Fulvestrant, the first FDA-approved selective estrogen receptor degrader, could overcome resistance associated with estrogen receptors modulators but is limited by poor oral bioavailability.^344,345^

ER-targeting PROTACs represent a significant advancement in targeted therapy for ER-positive breast cancers. These PROTACs have shown more potent degradation and enhanced anti-proliferative effects compared to fulvestrant.^346–349^ Three ER-targeting PROTACs are currently in clinical trials, with ARV-471 being the most advanced. In preclinical studies, ARV-471 demonstrated potent degradation with significant anti-proliferative effects, and a Phase I study reported good tolerability and a clinical benefit rate of 40% in patients with R/R advanced ER+/HER2- breast cancer.^350–352^ Ongoing Phase II studies are assessing higher doses, showing promising efficacy, especially in patients with ESR1 mutations.^352^ Encouraged by these preliminary data, ARV-471 entered two pivotal Phase III trials.

The clinical trials of ARV-471 and other oral PROTACs, including AC682 and SIM0270, are set to further validate their therapeutic efficacy, potentially reshaping the treatment landscape for breast cancer by overcoming resistance and offering more effective options for advanced cases.

#### Prostate cancer

Prostate cancer is one of the most common cancers among males globally, with AR playing a pivotal role in its pathogenesis. AR, a nuclear hormone receptor, drives the growth and survival of prostate cancer cells by mediating the effects of androgens.^353^ TPD, as a novel strategy for prostate cancer, degrades AR directly, and overcomes resistance. Initial efforts with peptide-based PROTACs faced challenges such as low degradation potency and poor cellular permeability.^354–356^ However, significant advancements began in 2008 with the development of small molecule AR degraders including PROTAC-A, which utilized an MDM2 inhibitor and a bicalutamide analog linked by a PEG-based linker.^355^ Although the efficacy was limited, it spurred the development of more effective small molecule AR degraders.^355,357,358^

By 2020, Takwale’s group had significantly advanced the field by developing TD-802, a novel CRBN binder. The DC_50_ of TD-802 was 12.5 nM, showcasing enhanced stability and tumor growth inhibition.^71^ AR-targeting PROTACs, such as ARCC-4 and ITRI-90, demonstrated superior efficacy in targeting mutated forms of AR and advanced into clinical trial for metastatic castration-resistant prostate cancer.^357,359,360^ ARV-766 and ARV-110^361,362^ are notable examples, achieving over 90% degradation of AR at nanomolar concentrations and are currently in Phase II clinical trials. Early results from the ARV-110 trial indicated significant antitumor efficacy, particularly in patients with specific AR mutations, where the PSA50 response rate was 46%, compared to 10% in wild-type patients.^363^

These developments indicate the feasibility of PROTACs for prostate cancer treatment, particularly for those with mutations resistant to conventional therapies. Ongoing and future clinical trials are expected to further refine the therapeutic applications and benefits of AR-targeting PROTACs.

#### Lung cancer

Lung cancer, particularly non-small cell lung cancer (NSCLC), remains one of the leading causes of cancer-related mortality globally.^364^ Epidermal growth factor receptor (EGFR), a cell surface tyrosine kinase receptor, is often mutated in NSCLC, leading to its persistent activation which drives the proliferation of tumor cells.^365^ Although small molecule inhibitors targeting EGFR significantly improve prognosis,^365–371^ resistance inevitably develops during treatment, commonly through secondary mutations such as EGFR T790M.^372^

PROTACs present a promising therapeutic strategy to overcome such resistance in NSCLC patients with mutant EGFR.^373^ These molecules, based on SMIs, effectively degraded diverse EGFR mutants such as EGFR Del19 and EGFR L858R at nanomolar concentrations, while sparing wild-type EGFR.^374–376^ Notably, Zhang et al. have developed an oral PROTAC, HJM-561, that specifically degraded the EGFR C797S triple mutants (Del19/T790M/C797S and L858R/T790M/C797S) in both CDX and PDX models.^377^ Furthermore, HSK-40118, another EGFR-targeting PROTAC, has shown considerable efficacy and is currently undergoing Phase I clinical trials to assess its efficacy in NSCLC patients with EGFR mutations. The ongoing research and clinical trials of EGFR-targeting PROTACs are crucial for further validation and may provide a new option for resistant NSCLC.

In addition, LYTAC and ATTEC have demonstrated capabilities for degrading EGFR. The cation-independent mannose-6-phosphate receptor (CI-M6PR, also known as IGF2R) is a typical LTR, which help transport lysosomal enzymes within cells by capping N-glycans with mannose-6-phosphate (M6P) residues.^247^ In 2020, Bertozzi’s group synthesized the LYTAC Ab-2,achieving 76% degradation of EGFR in dCas9-KRAB HeLa cells.^136^ Asialoglycoprotein receptor (ASGPR) is another LTR, highly expressed in hepatocytes and responsible for the clearance of glycoproteins through the process of clathrin-mediated endocytosis and subsequent lysosomal degradation. Triantennary N-acetylgalactosamine (tri-GalNAc) was a good ligand for ASGPR with low nanomolar affinity. In 2021, Bertozzi’s group reported GalNAc-LYTACs to degrade target such as EGFR and HER2.^378^ Their GalNAc-LYTACs effectively ablated EGFR and HER2 in HCC cells depending on the lysosomal system and the internalization of ASGPR.^378^ Moreover, in 2024, Xu et al. designed EGFR-ATTECs also using the LC3 ligand GW5074 to degrade EGFR, the result indicated that the ATTECs could induce EGFR degradation and exerted anti-proliferative effects with moderated safety.^379^ These technologies employ distinct mechanisms to target and dismantle EGFR, potentially offering new therapeutic avenues in oncology.

#### Pancreatic cancer

Pancreatic cancer, often termed the “king of cancers,” is notorious for its aggressive nature and dismal prognosis. It is characterized by rapid progression, late detection, and a notably short survival period.^380^ Current treatment strategies primarily involve surgery, chemotherapy, and radiation therapy.

##### Lactate dehydrogenase (LDH) degraders

Due to its late detection and resistance to conventional therapies, there is a critical need for innovative treatment approaches. LDH, an enzyme involved in the anaerobic conversion of pyruvate to lactate, plays a significant role in cancer metabolism, especially under hypoxic conditions common in pancreatic tumors. Elevated LDH levels are often associated with tumor aggressiveness and poor outcomes,^381–384^ making it a promising target for therapeutic intervention. Jin et al. introduced the first LDH-targeting PROTAC, MS6105, which successfully degrades both LDHA and LDHB isoforms. Notably, MS6105 shows enhanced anti-proliferative efficacy against pancreatic cancer cells compared to its parent SMIs.^385^ This breakthrough provides a promising new approach for the treatment of pancreatic cancer, warranting additional research to fully realize its therapeutic potential.

##### Phosphodiesterase delta (PDEδ) degraders

PDEδ is a prenyl-binding protein that assists in the transport and localization of the Kirsten rat sarcoma 2 viral oncogene homolog (KRAS) protein in cells.^386^ Targeting PDEδ in pancreatic cancer could disrupt this transport mechanism, and inhibit the oncogenic activity of KRAS, which is frequently mutated in this cancer type.^387^ Sheng’s group reported a series of ATTECs with GW5074 as the LC3 binder to degrade PDEδ, and the most promising compound **12c** caused the degradation of PDEδ via lysosome-mediated autophagy without interfering with PDEδ mRNA synthesis.^248^

Moreover, genes, frequently overexpressed in pancreatic cancer, such as KRAS, CDKN2A, TP53, and SMAD4,^388^ could serve as potential targets for PROTAC-based therapies in the treatment of pancreatic cancer.

#### Synovial sarcoma

Synovial sarcoma is a rare and aggressive type of soft tissue sarcoma that typically develops near the joints of the arms, neck, or legs. This cancer is defined by a specific chromosomal translocation that results in the formation of the SS18:SSX fusion protein.^389^ Bromodomain-containing protein 9 (BRD9), a non-BET bromodomain protein and a crucial component of the BAF complex, collaboratively supports the function of the SS18:SSX fusion protein across the genome in synovial sarcoma cells.^390^ Traditional inhibitors of BRD9 demonstrated only partial efficacy in inhibiting the growth of synovial sarcoma cells, while dBRD9-A, the BRD9 degrader, induced potent degradation of POI, cell cycle arrest and inhibition of tumor progression.^391^

In 2019, the Ciulli’s group advanced this approach by synthesizing a dual degrader for BRD7/9, systematically varying the conjugation patterns and linkers of an initially inactive compound to optimize its activity.^94^ More recently, DBr-1, a BRD9-targeting PROTAC based on the novel E3 ligase receptor DCAF1, was developed. This PROTAC offer a potential alternative treatment option for patients exhibiting resistance to VHL-based degraders.^392^ Furthermore, CFT8634, an oral degrader, has achieved potent and selective degradation of BRD9 with a DC_50_ of 2 nM, effectively impairing tumor cell growth in a dose-dependent manner both in vitro and in vivo.^393^ It has also demonstrated synergy with pomalidomide.^394^ Currently, two BRD9 degraders, CFT8634 and FHD-609, are undergoing clinical trials to elucidate their therapeutic potential and safety profiles.

### TPD in pan-cancer

In the field of oncology, numerous molecular targets play critical roles, not just in specific forms. These targets include key proteins and enzymes that regulate processes such as cell cycle progression, apoptosis, and metastasis. The broad applicability of these targets makes them ideal candidates for TPD strategies, which can be designed to selectively degrade these proteins in a wide range of cancers.

#### Kinase-related degraders

##### Anaplastic lymphoma kinase (ALK) degraders

ALK, a receptor tyrosine kinase, initially identified in anaplastic large cell lymphoma (ALCL),^395^ undergoes fusion gene rearrangements commonly observed in various tumors. This contributes to tumorigenesis via activating downstream signaling pathways, including PI3K/AKT, JAK-STAT, and MAPK.^396^ Currently, five SMIs have been approved by FDA for the treatment of NSCLC, which display potently and specifically anti-tumor efficacy, but drug resistance remains a challenge.^397,398^

In 2018, Powell et al. developed the CRBN-based ALK degraders, TL13-12 and TL13-112, achieving the degradation of ALK fusion protein in NSCLC, ALCL, and neuroblastoma cell lines. However, these degraders exhibited off-target effects and were less potent than their parent inhibitors in viability assays.^399^ Similar characteristics were observed in contemporaneous degraders MS4077 and MS4078.^400^ Kang et al. synthesized the VHL-based ALK degrader to realize the degradation of POIs and tumor inhibition. However, the efficacy comparison between degrader and its parent inhibitors had not been conducted in this study.^401^ Excitingly, a Brigatinib-degrader, SIAIS117, based on VHL ligands, effectively degraded the ALK protein, displaying slightly greater potency than Brigatinib. Notably, it retained anti-proliferative activity against cells transduced with ALK G1202R, providing a promising therapy for patients with this mutation.^11^ Subsequent studies further compare the efficacy between small molecule inhibitors and PROTACs. In 2021, Jiang et al. designed Alectinib-degrader SIAIS001, which exhibited superior cytolytic activity compared to Alectinib and good oral bioavailability.^402^ At the same year, Li et al. constructed B3 and validated it in vivo, which demonstrated improved anticancer activity compared to the parent inhibitor.^402^ In 2023, an oral degrader, CPD-1224, successfully degraded ALK L1196M/G1202R, slowing tumor growth in vivo, while the ALK inhibitor Lorlatinib had no effect.^403^

In the pursuit of enhanced precision, the novel folate-guided degrader was constructed for targeted delivery to reduce off-target toxicity.^404^ Crizotinib is the only ALK inhibitor approved in R/R ALCL patients with ALK-positive status. Despite its efficacy, a substantial proportion of patients relapse after drug withdrawal.^405^ Furthermore, some optimized ALK degraders exhibited efficacy in NSCLC,^406,407^ which may provide an alternative therapy for drug-resistant and R/R patients and resolve the conundrum of discontinuation.

##### AKT degraders

AKT, also known as protein kinase B, is a serine/threonine-specific protein kinase that plays a central role in the PI3K/AKT signaling pathway. Aberrant AKT signaling is commonly observed in cancers, leading to uncontrolled cell growth and survival. As a consequence, AKT is considered as a target for cancer research and therapeutic development.^408,409^ In 2020, Toker et al. constructed an AKT degrader, INY-03-041, consisting of the pan-AKT inhibitor GDC-0068 conjugated to lenalidomide. This compound induced sustained AKT degradation in various cancer cells and displayed notable anti-proliferative effects.^410^ Dong et al. devised a structurally unique AKT degraders, incorporating a 3,4,6-trisubstituted piperidine pharmacological warhead. This design promoted potent and selective degradation of AKT, and remarkable anti-proliferative effects in various hematological cancers. Moreover, combined with BTK inhibitor ibrutinib, B4 pronounced significant synergistic inhibition of proliferation in MCL cells.^411^ Based on structure-activity relationship studies, Jin et al. developed a series of AKT degraders, including MS21, that are more effective in degrading AKT in various tumor cells, including those with PTEN/PI3K mutations, compared to their parent SMIs. Their anti-tumor efficacy was also validated in a xenograft model.^412^ Furthermore, this group developed a new degrader, MS15, based on an AKT allosteric inhibitor, which also demonstrates AKT degradation and anti-proliferative activity in cancer cells with KRAS/BRAF mutations, offering a new approach to overcoming MS21 resistance.^412^

##### CDKs degraders

The CDK family, a subset of serine/threonine kinase subfamily, consists of 21 enzymes that play crucial roles in cell cycle regulation and transcription. Dysregulation of CDKs, particularly CDK1, 2, 3, 4, and 6, which orchestrate substrate phosphorylation to regulate cell cycle progression, and CDK7, 8, 9, and 11, which regulate transcription, is often implicated in uncontrolled cell division during neoplastic transformation.^413^ Although several CDKs inhibitors have been developed for cancer therapy, their clinical use is frequently limited by off-target effects.^414^ Consequently, PROTACs have emerged as a promising alternative, offering selective and potent degradation of CDKs.

Dual CDK4/CDK6 degraders displayed preferential degradation of CDK6.^415–417^ In 2019, Rao’s group^417^ constructed a PROTAC library based on dual CDK4/CDK6 inhibitors. The representative PROTAC CP-10 effectively degraded CDK6, regardless of its WT or mutant form, inhibiting hematopoietic cancer cell proliferation. These PROTACs exhibited selective CDK6 degradation and a preference for CRBN. Furthermore, Winter et al. highlighted the specific dependency of AML cell lines on CDK6, while CDK4 was less critical.^416^ Their CDK6-specific degrader, BSJ, exhibited rapid and potent degradation of CDK6. In addition, the preferential CDK6 degrader, YX-2-107, showed selective suppression of cell growth in Philadelphia chromosome-positive ALL, with reduced impact on normal hematopoietic progenitors and mitigated neutropenia.^415^

CDK2 implicated in blocking differentiation of AML cells,^418^ is challenging to target specifically due to highly similar ATP-binding sites with other CDKs.^419^ Rao’s group designed a potent and selective CDK2-targeting PROTAC CPS2, promoting cellular differentiation by degrading CDK2 in various myeloid/lymphoid cell lines without obvious toxicity.^420^ Moreover, Cheng et al. developed an orally available triple-target CDK 2/4/6 degrader, demonstrating potent degradation and effective induction of apoptosis in various cancer cells, particularly in malignant melanoma.^421^

Besides, CDK9 inactivation could reduce the expression of high turnover proteins like c-Myc and MCL-1, which are involved in regulating leukemia cell survival.^422^ Thus, CDK9 inactivation is considered valuable for AML therapy. BTX-A51, a multi-kinase inhibitor of CK1α and CDK9, has been approved for a Phase I clinical trial in relapsed or refractory AML.^423,424^ In 2019, the A51-based PROTAC PHM-A51 demonstrated potent degradation of CK1α and CDK9, along with anti-proliferative effects in AML and lymphoma cells at low nanomolar concentrations. Importantly, no obvious off-target effects were observed in peripheral blood mononuclear and fibroblast cells.^417^ In 2021, Bian et al. reported a PROTAC B03, specifically targeting CDK9, which demonstrated 20-fold more potent degradation of CDK9 than the warhead alone in MV4-11 cells.^425^ Research has also shown that CDK9-targeting PROTAC selectively degraded CDK9 in pancreatic cancer cells and enhanced their sensitivity to venetoclax.^426^

##### Neurotrophic receptor tyrosine kinase (NTRK) degraders

The NTRK genes encode the neurotrophic tyrosine receptor kinase family, which plays crucial roles in regulating cellular proliferation, differentiation, and apoptosis. NTRK gene fusions, prevalent across a variety of cancers, are recognized as a pan-cancer oncogenic factor.^427–432^ To combat these, the FDA has approved two SMIs, larotrectinib^433^ and entrectinib,^434^ and numerous other TRK inhibitors are currently under development.^435–440^ Despite their initial success, the development of acquired resistance has curtailed the efficacy of these treatments, highlighting the necessity for innovative therapeutic strategies.

In 2019, 51 compounds were synthesized which demonstrated the capacity to induce effective degradation of TRKC at concentrations between 1 and 10 μmol/L. This development provides a promising new approach for treating cancers characterized by NTRK fusions.^441^ In a significant advancement, the NTRK degrader CG001419 has recently entered clinical trials, potentially broadening the spectrum of treatment options for cancers driven by NTRK fusions.

##### B-Raf proto-oncogene, serine/threonine kinase (BRAF) degraders

The RAF family kinases, which function as the downstream of EGFR or the small GTPase RAS, are critical components of the MAPK signaling pathway (RAS–RAF–MEK–ERK).^442–444^ Mutations in RAS or RAF led to hyperphosphorylation of downstream targets, resulting in dysregulated signaling pathways that ultimately contribute to oncogenesis. Notably, the BRAF (V600E) mutation is prevalent across various cancers, making it a critical focus of contemporary research. Targeted inhibitors such as dabrafenib, vemurafenib, and encorafenib have been employed to treat these conditions. Although these agents have demonstrated antitumor activity, resistance often develops due to secondary mutations in RAF.^445,446^

To address these challenges, researchers from the University of Toronto have developed a PROTAC molecule, P4B, based on the inhibitors dabrafenib and BI 882370, targeting BRAF (V600E). P4B uniquely degrades the BRAF (V600E) protein more specifically than traditional SMIs and remains effective against cells harboring V600D and G466V mutations. However, RAS activation can induce the dimerization of BRAF (V600E), thereby reducing P4B’s efficacy.^447^ Future therapies of maintaining BRAF (V600E) in a monomeric state may expand the clinical applicability of P4B.

CFT1946, an oral PROTAC, demonstrated promising anti-tumor activity in BRAF (V600X) preclinical models. Notably, in combination with cetuximab, CFT1946 demonstrated superior activity compared to the standard of care combination of SMIs with cetuximab, in all colorectal cancer models.^448^ Based on the preclinical profile, CFT1946 is currently being evaluated in a Phase I trial.

#### Epigenetic regulation-related degraders

##### Bromodomain containing 4 (BRD4) degraders

BRD4, a member of the BET protein family, serves as an epigenetic ‘reader’ by regulating gene transcription, chromatin remodeling, and transcriptional activation. These proteins recognize and bind to acetylated lysine residues on histone proteins via their two tandem N-terminal bromodomains, BD1 and BD2.^449^ The BET protein family includes BRD2, BRD3, BRD4, and bromodomain testis-specific protein, with BRD4 being the most extensively studied. It plays critical roles in DNA damage repair, telomere regulation, and the expression of proto-oncogenes such as c-MYC and BCL-2.

Despite the promising efficacy of BET inhibitors, challenges such as drug resistance,^450–452^ toxic side effects, and feedback upregulation of BRD4 remain significant hurdles.^453^ In 2015, the first BET degrader, dBET1, achieved complete degradation of BRD4 in AML cell lines and demonstrated significant in vivo antitumor effects, highlighting potential advantages over traditional BET inhibitors.^67^ The same year, Crews et al. developed ARV-825, which featured a novel linker modification compared to dBET1 and showed potent BRD4 degradation in BL cells.^454^ In addition, Hu et al. explored the role of BRD4 isoforms through warhead modification, leading to the synthesis of a range of BET degraders that selectively degrade specific BRD4 isoforms, thereby inducing distinct biological activities related to cell cycle regulation and apoptosis.^455^

Utilizing PG analogs, Li et al. developed SJ995973, which exhibited exceptional stability and effective degradation of BRD4 at low picomolar concentrations in MV4-11 cells.^73^ In addition, they further enhanced the cell permeability of PROTACs in AML cell lines through an amide-to-ester substitution approach.^456^ Currently, RNK05047, a BRD4 degrader, has entered a Phase I/II clinical trial for advanced solid tumors and lymphomas.

AUTACs have shown significant efficacy in degrading the BRD4 protein. In 2019, Arimoto et al. utilized JQ1 as the warhead in AUTAC3 to target BRD4, but found it was less effective at degrading the nuclear protein. Subsequently, Ouyang et al. designed another series BRD4-targeting AUTAC, inducing autophagy degradation of BRD4 protein by tethering LC3. AUTAC 10f showed the most potent activity by attaching the LC3 warhead GW5074 to the BRD4 ligand JQ1 using a PEG linker.^244^ Treated with 20 μM 10f for 24 h, BRD4 degradation in HeLa cells reached 92%, over 80% in several TNBC cells, 99% in MDA-MB-231 cells. The results showed that AUTAC 10f can target LC3 and degrade BRD4 through autophagy.^244^ The positive outcomes demonstrate that AUTACs are viable for discovering autophagy-related drugs. However, the working mechanism of AUTACs should be deeply understood. For example, their use in removing protein aggregates remains untested.

To extend the capabilities of BET degraders, additional E3 ligases such as DCAF15 and FEM1B have been utilized.^81,95^ Moreover, innovative BET-targeting PROTACs, including Macro PROTAC, CLIPTAC, Photo-PROTAC, and antibody–drug conjugates, have been synthesized to improve drug properties and selectivity.^108,318,457–459^

##### Polycomb repressive complex 2 (PRC2) degraders

PRC2 is composed of three core subunits: enhancer of zeste homolog 2 (EZH2), suppressor of zeste 12 (SUZ12), and embryonic ectoderm development (EED). This complex serves as an epigenetic modulator of transcription, regulating gene expression through the methylation of H3K27. Its dysregulation is linked to various cancers, with EZH2 interacting with oncogenes to promote tumorigenesis through both canonical and noncanonical pathways.^460^ Although EZH2 inhibitors are widely used, their effectiveness is limited by off-target effects and the development of resistance.^461,462^

In recent years, significant progress has been made in the development of PRC2-targeting degraders and PROTACs. In 2020, Bloecher’s group^463^ and James’s group^464^ designed VHL-based degraders targeting EED, which induced rapid protein degradation but exhibited comparable or inferior anti-proliferative activities compared to SMIs in B lymphoma cells with EZH2 mutation. In 2021, Yu et al. reported a series of EZH2-targeting PROTACs, including E7, which mediated a decrease of PRC2 subunits and demonstrated superior inhibition of tumor growth compared to SMIs in DLBCL cells, irrespective of EZH2 mutation status. Moreover, E7 downregulated EZH2-mediated downstream genes, suggesting its ability to eliminate the nonenzymatic oncogenic role of EZH2.^465^ This superior therapeutic activity of EZH2 degraders, YM181, over inhibitors was validated in a xenograft mouse model using the SU-DHL-6 cell line.^466^ Moreover, a series of VHL-based EZH2 PROTACs were developed, among which MS8815, featuring a longer linker, almost completely degrades EZH2 in triple-negative breast cancer cells. It demonstrated stronger anti-proliferative effects than YM281. In 2022, Wang et al. revealed the noncanonical oncogenesis pathway of EZH2 in AML, explaining the limited antitumor effect of SMIs. Furthermore, they developed the EZH2 degrader, MS177, which achieved effective on-target depletion of EZH2 and c-MYC, demonstrating potent anti-tumor activity in both in vivo and in vitro.^467^ James’ group reported the second-generation EED degrader, UNC7700, featuring an optimized cis-cyclobutane linker,^468^ which displayed enhanced anti-proliferative effects and degradation activity compared to its predecessor, UNC6852.^464^ The combination of EZH2 degraders with dual EGFR/HER2 inhibition induced apoptosis and cell cycle arrest, providing a promising therapeutic approach for BL.^469^ Moreover, Li et al. explored the activity of EZH2-targeting PROTACs based on four common ligands and identified MDM2 as the most active molecule.^470^ These advancements enhance the potential for more targeted and effective cancer therapies by addressing the dysregulation of PRC2 components, particularly EZH2.

#### Apoptosis-related degraders

##### Ubiquitin-specific protease 7 (USP7) degraders

USP7 is a deubiquitinating enzyme that stabilizes target proteins through deubiquitination. This activity is implicated in various cellular functions depending on target proteins.^471–473^ The relationship between USP7 and p53 is intricate and dynamic. While stabilizing p53 has tumor-suppressive effects, USP7 also functions in stabilizing MDM2, which, in turn, inhibit the function of p53.^474^ Steinebach et al. designed the USP7 degrader, PROTAC 17 (CST967), degrading USP7 and upregulating p53 levels. Moreover, PROTAC 17 decreased viability of MM.1S cells.^475^ Zhou et al. further investigated the function of USP7 degrader for p53. The research supported the notion that U7D-1, the USP7 degrader, impeded cell growth and elevated p53 levels through a partially p53-dependent mechanism in cells lacking TP53 mutation. In TP53 mutant cells, USP7 exhibited anti-proliferative effects by activating the apoptotic and E2F pathways, distinguishing it from inhibitors with no impact on cell growth.^476^ These findings highlight the potential of USP7 degraders as a therapeutic approach for modulating the intricate interplay between USP7 and p53 in cancer treatment.

##### p53 degraders

The transcription factor, p53, pictorially called “guardian of the genome”, repairs DNA damage and induces apoptosis in mutant cells as a tumor suppressor.^477^ However, TP53 gene mutations are commonly observed in cancers, resulting in the loss of pro-apoptotic functions and enabling the proliferation of tumor cells. Thus, p53 has emerged as an appealing target for cancer therapy. However, no effective related drugs have been approved yet due to its lack of hydrophobic binding pocket and inhibition activity. Currently, therapeutic strategies for p53 focus on inhibiting mutant p53 and reactivating WT p53 by blocking negative regulators, notably MDM2. In 2023, Xie et al. developed dp53m-RA, the first p53-R175H degrader designed to target the most common hotspot TP53 mutation. This degrader, employing an RNA aptamer, effectively inhibited proliferation in various lung and breast cancer cells with p53-R175H mutation, while leaving wild-type p53 and other p53 mutants unaffected and promoting p53 downstream effectors.^470^ Moreover, the reactivated p53 was observed in PROTACs targeting MDM2 or BRD4, as discussed in the relevant section.

##### c-MYC-targeting PROTACs

The oncogene c-MYC is an essential transcriptional regulator, modulating multiple cellular processes such as cell proliferation and apoptosis.^478^ Dysregulation of c-MYC is a common occurrence in various tumors, often acting downstream of oncogenic signaling pathways. Targeting c-MYC for therapy development is indeed challenging due to the complex nature of this transcriptional regulator. In a groundbreaking development, Andreeff’s group developed the novel dual c-MYC/GSPT1 degrader, GT19715, which potently degraded both c-MYC and GSPT1. This resulted in tumor growth inhibition while leaving normal cells unaffected. Interestingly, they found that Venetoclax-resistant cells which overexpress c-MYC and GSPT1, exhibited heightened sensitivity to GT19715.^479^ To date, the most commonly used approach to block c-MYC involves targeting its transcriptional co-regulators, such as BRD4, instead of directly targeting c-MYC. C-MYC direct-targeting PROTACs represent a potentially paradigm-shifting strategy for the treatment of MYC-driving cancers.

#### Others

##### IKZF2 degraders

IKZF2 (also known as Helios), a zinc-finger transcription factor, is pivotal in regulating immune homeostasis and identified as a potential immunotherapeutic target through structural studies.^480,481^ Particularly relevant to AML, IKZF2 influences the leukemic stem cells (LSCs) by promoting self-renewal and inhibiting myeloid differentiation.^482^ The targeted degradation of IKZF2 emerges as a rational therapeutic strategy for myeloid leukemias.

NVP-DKY70 demonstrates dose-dependent and selective degradation of IKZF2 (maximum degradation 69%, DC_50_ 11 nM), while sparing IKZF1/3, in AML cell lines. It not only inhibited tumor growth in patient-derived xenografts mice but also modulated immune responses by reducing Treg cell suppression and enhancing Teff cell functions in cynomolgus monkeys. Interestingly, following PD1 blockade, Treg cells showed increased IKZF2 protein levels. However, when combined with the PD1 monoclonal antibody PDR001, the therapy did not surpass the efficacy of monotherapy in preclinical settings. The efficacy of this combination is currently being evaluated in a Phase I clinical trial for solid tumors.^483^ Another MG, PRT-101, induces rapid and robust degradation of IKZF2 at sub-nanomolar concentrations, and displayed superior pharmacokinetics and antitumor efficacy in vivo compared to DKY709.^484^ In addition, dual degraders targeting both IKZF2 and CK1α have shown potential in promoting myeloid differentiation and inhibiting AML progression in vivo, highlighting new avenues for AML treatment.^311^ These findings underscore the promise of novel IKZF2 degraders, characterized by rapid absorption and favorable bioavailability, as immunotherapeutic agents for treating tumors.

##### G1 to S Phase transition 1 (GSPT1) degraders

GSPT1, a crucial target in cancer therapy, plays a significant role in cell cycle regulation and apoptosis.^485^ Several GSPT1 degraders are currently under clinical evaluation.

CC-90009, a novel CELMoD, emerged as the first MG from BMS’s library via phenotypic screening.^486^ In preclinical studies, it selectively degraded GSPT1, exhibiting strong anti-proliferative effects in AML cell lines and patient-derived cells. CC-90009 effectively induced the integrated stress response pathway, particularly targeting LSCs, and promoted myeloid differentiation in AML progenitor cells, offering a novel approach to eradicating AML.^487^

In clinical application, a Phase I trial of CC-90009 in patients with R/R AML indicated profound GSPT1 degradation with notable responses including CR and morphologic CR with incomplete blood count recovery.^488^ Notwithstanding the occurrence of significant treatment-related adverse events (TRAEs) such as hypocalcemia and hypotension, these were manageable with preemptive dexamethasone administration.^489^ Ongoing Phase I/II trials are evaluating CC-90009 in combination with venetoclax and azacitidine, promising to expand its therapeutic impact.^490^ Mechanistic studies revealed that CC-90009 affects AML progenitor cells and LSCs by modulating complex signaling pathways. Disruptions in the ILF2 and ILF3 complex or the TSC1 and TSC2 genes affect the degradation efficacy of CC-90009 by altering CRBN expression and GSPT1 binding, respectively. The degradation of GSPT1 triggers the integrated stress response pathway, involving key proteins such as GCN1, GCN2, and ATF4, inducing apoptosis in AML cells. These findings enhance our understanding of CC-90009’s mechanisms and its potential for broader clinical applications.^491^

MRT-2359 is especially effective in MYC-dependent cell lines and has shown preferential activity in preclinical models of NSCLC and SCLC that express high levels of N- and L-MYC. It also exhibited antitumor effects in neuroendocrine lung cancers and lymphoma patient-derived xenografts.^492^ MRT-2359 is undergoing a Phase I/II clinical trial to evaluate its efficacy in treating MYC-driven cancers such as NSCLC, SCLC, high-grade neuroendocrine cancers, and diffuse large B-cell lymphoma (NCT05546268). This strategy explores the indirect degradation of MYC via GSPT1 inhibition, potentially addressing the therapeutic limitations of direct MYC targeting.

BTX-1188, an orally bioavailable MG, has exhibited substantial efficacy in preclinical studies by degrading key proteins, such as GSPT1, IKZF1/3, and CK1α. By degrading IKZF1/3, BTX-1188 reduced pro-inflammatory cytokine production, mitigating the systemic inflammation triggered by GSPT1 degradation and thus broadening its therapeutic window. BTX-1188 is about 100 times more potent than CC-90009, highlighting its potential to overcome treatment resistance. Its antitumor effects have been confirmed in AML patient-derived cells resistant to standard treatments.^493^ Currently, BTX-1188 has entered Phase I clinical trials for advanced solid tumors and AML, however, the current status is still unclear. These developments suggest a growing interest in exploiting GSPT1 as a therapeutic target across different cancer types, with the potential to significantly enhance treatment outcomes.

##### KRAS degraders

KRAS, a small GTPase, cycles between a GTP-bound active state and a GDP-bound inactive state, driven by GTP hydrolysis and nucleotide exchange.^494^ KRAS mutations are among the most common oncogenic alterations in cancer,^495^ leading to the constitutive activation of downstream pathways such as MAPK and AKT-mTOR, which are crucial for cell proliferation and survival. SMIs targeting KRAS mutations, specifically KRAS G12D and G12C, have been developed, yet challenges such as acquired resistance including secondary KRAS mutations and abrogated feedback reactivation necessitate novel therapeutic approaches.^496,497^

In contrast to traditional SMIs, PROTACs have emerged as a robust alternative to achieve more durable and potent therapeutic outcomes. In 2020, LC-2, the first endogenous KRAS-targeting PROTAC, was introduced. It effectively degrades KRAS G12C and disrupts MAPK signal transmission, offering a new strategy for treating cancers driven by KRAS mutations.^13^ In addition, compound 8o has demonstrated significant efficacy in degrading various KRAS mutants (including G12C, G12V, G12S, G12R, G13D), while sparing wild-type KRAS. This activity leads to the inhibition of downstream pERK activation, showcasing antitumor effects in cancer mouse models.^498^ Remarkably, ASP3082 targets KRAS G12D for degradation, thereby inhibiting downstream molecular signaling and apoptotic respons, and displays dose-dependent antitumor activity across multiple cancer models with the KRAS G12D mutation.^499^ A Phase I clinical trial is currently ongoing to evaluate this innovative treatment.

##### Signal transducer and activator of transcription 3 (STAT3) degraders

STAT3 is a transcription factor that regulates genes essential for various biological processes.^500^ Abnormal activation of STAT3 is frequently associated with cancer,^501^ positioning it as a critical target for oncological therapies, despite the absence of a targetable active binding site. In 2019, Bai et al. engineered SD-36, the first potent and selective STAT3 degrader, through comprehensive optimization. This degrader swiftly reduced STAT3 levels, inhibiting the growth of leukemia and lymphoma cell lines. In vivo studies demonstrated complete tumor regression in mouse xenograft models of AML and anaplastic large cell lymphoma, with SD-36 displaying well-tolerated effects, underscoring its therapeutic potential.^502,503^

In addition, SD-91, a hydrolysis product of SD-36, sustained effective protein depletion and tumor regression in the MOLM-16 xenograft model with weekly administration.^504^ These findings highlight the consistent anti-tumor activity of the compound over extended periods. In the Phase Ia/b trial, KT-333 showed robust and dose-dependent degradation of STAT3, with mean maximum degradation exceeding 60% across varying dosages. This trial also demonstrated KT-333’s preliminary safety and efficacy. Remarkably, after two treatment cycles, one patient achieved PR, and no serious TRAEs were reported, emphasizing the safety profile of KT-333.^505^ These advancements in STAT3-targeting PROTACs exemplify significant strides in the development of new cancer therapies, particularly in effectively targeting transcription factors which are previously deemed as challenge due to the lack of conventional binding sites.

##### Programmed death-ligand 1 (PD-L1) degraders

PD-L1 functions as an immune checkpoint molecule that suppresses T cell activity by binding to its receptor PD-1, aiding tumor cells in evading immune surveillance.^506^ Targeted therapies against PD-L1 have become a critical component in the treatment regimens for various cancers.^507^ Recently, researchers have begun to explore TPD approaches to degrade PD-L1, a novel strategy that shows potential to enhance antitumor immunity by degrading the PD-L1 protein instead of merely blocking signaling pathways. Yang et al. developed a new PROTAC compound, 21a, which significantly reduced PD-L1 protein levels in MC-38 cancer cells, thereby enhancing the infiltration of CD8 + T cells and inhibiting the in vivo growth of MC-38.^508^ Moreover, in 2020, Bertozzi’s group synthesized LYTAC Ab-3, incorporating polyclonal anti-mouse IgG and azide-terminated M6Pn glycopolypeptides via copper-free strain-promoted azide–alkyne cycloaddition. After 36 h of Ab-3 treatment, PD-L1 degradation reached 50%.^136^ In 2023, Liu et al. reported a CI-M6PR and PD-L1 dual-specificity targeting LYTAC, synthesized by DBCO-modified DNA, which enhanced the specific binding to PD-L1 through biorthogonal covalent conjugation. This covalent LYTAC exhibited longer retention on PD-L1 and led to more extensive degradation than its noncovalent counterpart.^509^ These studies demonstrate the potential of developing innovative protein degradation tools targeting PD-L1 using various chemical and biological strategies.

### TPD in neurodegenerative diseases

With the acceleration of global aging, central nervous system disorders such as Alzheimer’s disease (AD) and Parkinson’s disease (PD) have increasingly become a significant public health burden.^510^ Current treatments for these disorders primarily rely on pharmacological interventions and symptom management, yet these approaches often fail to halt disease progression.^510,511^ Moreover, many traditional drugs face challenges such as poor penetration of the blood-brain barrier (BBB) and significant side effects, limiting their therapeutic efficacy.^512^ Many neurodegenerative disorders are characterized by the accumulation of aggregated proteins. TPD leverages the cell’s natural degradation mechanisms to selectively degrade and eliminate pathogenic proteins, providing a promising therapy for neurodegenerative diseases.

#### AD

AD is a neurodegenerative disorder characterized by memory decline, cognitive impairment, and behavioral changes. The pathogenesis of AD involves multiple pathological processes, including the abnormal accumulation of amyloid-β forming amyloid plaques^513^ and the pathological hyperphosphorylation of tau protein forming neurofibrillary tangles.^514^ These pathological changes ultimately lead to neuronal dysfunction and death. AD treatments primarily involve symptomatic drugs, such as acetylcholinesterase inhibitors and NMDA receptor antagonists.

TPD technology, capable of precisely degrading pathogenic proteins, holds promise in this context. Li et al. were the first to design a series of Tau-targeting PROTAC peptides, which degraded tau protein levels in the brains of AD mouse models. However, due to the BBB limitations, these peptides only function via intranasal and intravenous administration.^515^ Haggarty et al. building on the tau positron emission tomography tracer 18F-T807, developed the small molecule tau degrader QC-01–175, which preferentially degrades tau in neuronal cell models derived from patients with frontotemporal dementia.^516^ Excitingly, Wang et al. synthesized a low-toxicity tau-targeting PROTAC that achieved potent degradation of tau (IC_50_ = 5 nM) through intracerebral and subcutaneous administration in hTau-transgenic and 3×Tg-AD mouse models, improving synaptic and cognitive functions. Although oral administration is the most ideal, gavage did not result in reduced tau, suggesting the need for further structural optimization. In addition, glycogen synthase kinase 3 (GSK-3), a highly conserved serine/threonine kinase, induces the hyperphosphorylation of tau, playing an important role in NFT formation.^517,518^ Beyond direct tau degradation, a potent GSK-3 degrader named PT-65 was developed based on a click chemistry platform. The degradation of GSK-3 effectively reduced tau hyperphosphorylation and ameliorated learning and memory impairments in animal models.^519^ p38 mitogen-activated protein kinase is considered as cell proliferation, differentiation, apoptosis, and inflammation.^520^ Recently, a study indicated that inhibition of p38 could alleviate pathological symptoms of AD, Son et al. reported a PROTAC, PRZ-18002, which could induce degradation of phospho-p38 protein and p-38, and the drug could reduce tau protein levels in the hippocampus of 5×FAD mice.^521^ These findings underscore the potential of TPD technology in treating neurodegenerative diseases by targeting specific molecular markers.

#### PD

PD is a chronic neurodegenerative disorder characterized primarily by motor symptoms including tremor, rigidity, bradykinesia, and postural instability. The pathogenesis of PD is associated with the gradual loss of dopaminergic neurons, particularly in the substantia nigra.^522^ Current treatment strategies for PD primarily rely on pharmacological therapies, such as levodopa and dopamine receptor agonists, which effectively alleviate early symptoms. However, as the disease progresses, the efficacy of these drugs diminishes, they may lead to side effects such as motor fluctuations and drug-induced dyskinesias.^523^

##### α-Synuclein (α-Syn) degraders

α-Syn is primarily expressed at presynaptic terminals, where it regulates dopamine release and reuptake, crucial for maintaining neurotransmission balance.^524^ However, in PD, α-Syn abnormally aggregates, forming what are known as Lewy bodies, which disrupts normal cellular functions, leading to neuronal damage and death.^525^ Pang et al. synthesized a series of degraders based on the α-Syn inhibitor sery384 through silico docking studies, effectively promoting its degradation.^526^ This provides a new approach for α-Syn-related neurodegenerative diseases. Based on Anle138b, lenalidomide, and pomalidomide, Seneci et al. designed a series of α-Syn degraders, utilizing click chemistry. They confirmed the safety and efficacy of these degraders in reducing α-Syn aggregation in iPSC-derived dopaminergic neurons with four copies of the α-Syn gene, as well as in patient-derived dopaminergic neurons.^527^ Given the interplay of proteins associated with neurodegenerative diseases, Pang et al. further designed and synthesized a series of dual PROTACs targeting both α-Syn and tau. Encouragingly, these degraders crossed the BBB and effectively degraded tau in cellular and PD mouse models, protecting dopaminergic neurons from damage.^528^

##### Leucine-rich repeat kinase 2 (LRRK2) degraders

LRRK2 is a multifunctional protein with dual enzymatic activities as both a kinase and GTPase. Studies have shown that mutations of LRRK2 enhance the aggregation of α-synuclein within Lewy bodies, promoting neurodegenerative changes.^529–531^ Thus, degrading LRRK2 protein represents a promising approach for treating PD. Dömling explored the development of LRRK2 degraders. Although the synthesized PROTACs demonstrated good cellular permeability and target binding, they failed to achieve effective degradation.^532^ Following Dömling’s initial attempts, Ciulli et al. developed XL01126, the LRRK2 degrader, through two rounds of screening, which effectively degraded both mutant and wild-type LRRK2. Notably, XL01126 exhibited high cellular permeability and could cross the BBB in mouse models via oral or enteral administration.^533^ However, despite its strong therapeutic potential, XL01126 has not yet been validated for improving PD symptoms. Recently, Arvinas announced the first-in-human dosing of ARV-102, which could cross the BBB and degrade LRRK2. In non-human primates, orally administered ARV-102 could reach deep-brain regions and degrade LRRK2 by nearly 90%. The Phase 1 clinical trial for ARV-102 has commenced in the Netherlands.

In summary, these studies indicate that the development of LRRK2 degraders provides a new direction for PD treatment. However, further research is necessary to validate their clinical efficacy in symptom improvement.

#### Huntington’s disease (HD)

HD is a hereditary neurodegenerative disorder characterized by progressive motor dysfunction, cognitive decline, and psychiatric symptoms.^534^ The pathogenesis of HD is primarily linked to the abnormal expansion of the huntingtin protein (HTT), which leads to protein dysfunction and gradual neuronal damage.^535^ Current treatments for HD focus mainly on managing symptoms with antipsychotic and antidepressant medications, which alleviate psychiatric and motor symptoms but do not halt disease progression or slow the degenerative process. Ishikawa et al. designed two hybrid small molecules based on ligands for cIAP1 and probes for mutant HTT (mHTT). These synthetic compounds effectively reduced mHTT levels in fibroblasts from HD patients, suggesting a novel therapeutic approach that targets the underlying molecular pathology of HD.^536^ Besides, mHTT can be degraded through autophagy by associating with autophagosomal protein LC3.^249,537^ Several mHTT-LC3 linker compounds (ATTECs) that facilitate allele-selective degradation of mHTT have been identified.^249^ These compounds specifically target mHTT for degradation through autophagy, improving HD symptoms in fly and mouse models. This study validates the potential of lowering mHTT with ATTEC as a new treatment strategy for HD. Increasing evidence indicates the success of ATTEC. These advancements represent a significant progress towards developing treatments that not only manage symptoms but also modify the disease course.

#### Stroke

Stroke, a significant medical condition, arises when the blood supply to parts of the brain is blocked or reduced, leading to potential permanent damage or death of brain cells. Wang et al. designed a series of CMA-base degraders to knock down native neuronal proteins using the cell membrane-penetrating sequence TAT. They found that the peptide degraders quickly and effectively reduced the expression levels of death-associated protein kinase 1, postsynaptic density protein 95, and α-Syn in a rapid, reversible, and dose-dependent manner. This study was the first to verify the concept of a CMA-based degrader.^253^ The results confirmed that the target peptide can cross the BBB and knock out the protein of interest in both primary neurons and rat brains. Zhu et al. developed another CMA-based degrader (TAT-CDK5-CTM peptide), which can penetrate cell membranes and showed in a mouse stroke model that it disrupts the CDK5-NR2B interaction, causing CDK5 degradation linked to stroke.^538^ Significant advancements have been made in elucidating the role of CMA in neurodegenerative disorders. However, the mechanisms of CMA are not fully understood, and the effectiveness of CMA-based degraders largely depends on penetrating peptides like TAT. This makes their therapeutic effectiveness heavily reliant on the delivery efficiency of these peptides.

### TPD in metabolic disorders

Metabolic syndrome is marked by high blood pressure, dyslipidemia, elevated glucose, and obesity, increasing the risk of diseases like diabetes and cardiovascular disorders.^539,540^ These conditions are linked to metabolic imbalances involving proteins and fats. Targeting enzymes and receptors involved in these processes could effectively manage this syndrome. TPD has shown promising therapeutic effects in metabolic diseases.

#### Lipid-lowering TPDs

##### HMG-CoA reductase (HMGCR) degraders

HMGCR is the key enzyme in cholesterol synthesis and a primary target for statins,^541^ which reduce cholesterol by binding to the active site of HMGCR.^542,543^ Despite their effectiveness, statins sometimes cause side effects such as muscle damage and insulin resistance.^544–548^ To address these challenges, new PROTAC molecules have been developed targeting HMGCR. For instance, Rao et al. created a PROTAC by linking atorvastatin with pomalidomide (P22A), effectively degrading HMGCR and reducing cholesterol synthesis in Huh7 cells.^541^ Similarly, Zhu et al. developed an oral PROTAC (21b and 21c) by combining a VHL ligand with lovastatin acid, which showed enhanced degradation of HMGCR and greater cholesterol reduction in hypercholesterolemic mice compared to lovastatin acid alone.^549^

These advancements in TPD offer promising new pathways for treating lipid metabolism disorders and associated conditions like coronary heart disease by selectively degrading crucial metabolic enzymes.

##### Patatin-like phospholipase domain-containing 3 (PNPLA3) degraders

PNPLA3, also known as adiponutrin, plays a key role in fat metabolism in the liver and adipose tissue by hydrolyzing triglycerides and retinyl esters.^550^ Genetic variations in PNPLA3 significantly contribute to the development of fatty liver disease (FLD) due to its involvement in hepatic lipid metabolism.^551,552^ BasuRay et al. observed that the accumulation of PNPLA3 on lipid droplets leads to steatosis. To address this, they developed a novel degrader, PROTAC3, which combines a VHL ligand with chloroalkane and a modified bacterial dehalogenase. This degrader effectively degrades PNPLA3, improving FLD symptoms and offering a new therapeutic approach for the disease.^553^

##### Liver X receptors α and β (LXRα and LXRβ) degraders

LXR, specifically LXRα and LXRβ, are nuclear receptors that are pivotal in regulating inflammation as well as cholesterol, fatty acid, and glucose metabolism.^554^ LXR agonists have been identified as potential cholesterol-lowering agents useful in the treatment of atherosclerosis, diabetes, and Alzheimer’s disease.^555^ However, the activation of LXR has been linked to the promotion of hepatic steatosis.^556^ As a complementary strategy, Demizu et al. developed a series of agonist-based LXRβ PROTACs, achieving the targeted degradation of LXRβ in HuH-7 cells.^557^ Nevertheless, the effectiveness of this approach in vivo and its impact on specific diseases still requires further validation.

##### Sterol regulatory element-binding proteins (SREBPs) cleavage activating protein (SCAP) degraders

SCAP, a crucial membrane protein in the endoplasmic reticulum, functions as an escort and activator for SREBPs, transporting them to the Golgi apparatus for activation when cellular cholesterol is low. This process increases gene expression for cholesterol and lipid synthesis.^558,559^ Researchers have found that reducing SCAP levels can enhance lipid clearance and reduce oxidative stress, thus helping prevent atherosclerosis.^560^ Zhang et al. discovered that lycorine promotes SCAP degradation via a novel lysosomal pathway by enhancing SCAP’s interaction with SQSTM1/p62. This pathway has been termed SQSTM1-mediated autophagy-independent lysosomal degradation (SMAILD). Remarkably, lycorine not only reduced obesity, hyperlipidemia, hepatic steatosis, and insulin resistance in a high-fat diet mouse model but also outperformed lovastatin, offering new insights into modulatory compounds in non-proteasomal systems.^561^ Further exploration of the SMAILD pathway is needed to enhance our grasp of its mechanisms.

##### Proprotein convertase subtilisin/kexin type 9 (PCSK9) degraders

PCSK9, an enzyme crucial in cholesterol metabolism, binds to low-density lipoprotein receptors on hepatocyte surfaces, reducing the liver’s ability to remove low-density lipoprotein cholesterol (LDL-C).^562^ Elevated levels of LDL-C are closely associated with an increased risk of cardiovascular diseases, making PCSK9 a significant target for lipid-lowering therapies.^563^ The use of PCSK9 inhibitors has been revolutionary in managing hypercholesterolemia.^564^ However, these treatments are costly and necessitate frequent injections, highlighting the demand for more accessible therapeutic alternatives. Purohit et al. have innovatively applied computational screening methods to design a PROTAC targeting PCSK9, presenting a new avenue for PCSK9 degradation therapy.^565^ However, while effective, these treatments are costly and require frequent dosing through injections, highlighting the need for more accessible therapeutic alternatives.

##### Estrogen-related receptor alpha (ERRα) degraders

ERRα, a nuclear receptor, is critical for managing gene networks that regulate energy homeostasis, encompassing fat and glucose metabolism, mitochondrial function^566^ and muscle and bone development.^567^ Identified as a potential therapeutic target, ERRα holds promise for treating diabetes^568^ and osteoporosis.^569^ Carnosic acid has been proved to be a dual-target drug that degrades ERRα and SREBP2, effectively reducing cholesterol levels and inhibiting bone loss.^570^ In addition, PROTACs targeting ERRα have been developed that significantly degrade this protein in vitro and in vivo.^28,571^ However, further evaluation is needed to assess the effectiveness of this approach in reducing LDL-C and its potential impact on atherosclerosis.

##### Targeted protein tripartite motif containing 24 (TRIM24) degraders

TRIM24, an E3 ubiquitin ligase from the TRIM protein family, is associated with the onset and progression of various cancer types. The TRIM24 degrader, dTRIM24, has shown potent anti-tumor effects in preclinical trials for glioblastoma and AML.^572,573^ In addition, TRIM24 plays a critical role in immune regulation by inhibiting the acetylation of STAT6, which promotes the polarization of macrophages towards the M2 phenotype. This action is essential for anti-inflammatory responses.^574^ In contrast, pro-inflammatory M1 macrophages are crucial in atherosclerosis development and prevalent in atherosclerotic plaques, contributing to chronic inflammation.^575,576^ Interestingly, M2 macrophage-derived exosomes can induce phenotypic switch from M1 to M2 type during wound healing,^577^ suggesting that the M2 macrophage membranes may function similarly to exosomes and target M1 macrophages in atherosclerotic plaques. Building on this concept, Zhang et al. developed a sophisticated strategy by encapsulating the TRIM24 degrader, dTRIM24, within PLGA nanoparticles coated with M2 macrophage membranes. This design ensures the responsive release of dTRIM24 in the acidic environment generated by inflammatory M1 macrophages, facilitating the degradation of TRIM24 in these cells and their subsequent shift towards the M2 phenotype. This targeted delivery method effectively reduces plaque formation in a mouse model of atherosclerosis, demonstrating the precise targeting capabilities of this drug delivery system.^578^

This advanced approach not only highlights the therapeutic potential of PROTACs in managing complex diseases but also marks a significant progression in targeted drug delivery technologies, offering more efficient and specific treatment options for chronic inflammatory conditions.

#### Blood glucose-lowering TPDs

Protein Tyrosine Phosphatase 1B (PTP1B), a phosphatase enzyme, regulates various signaling pathways by dephosphorylating specific proteins. Specifically, PTP1B can dephosphorylate the insulin receptor and its substrates, negatively regulating insulin signaling.^579^ Consequently, it is considered a potential target for treating diabetes, obesity.^580,581^ Various PTP1B inhibitors were synthesized, but most of the trials have been terminated due to poor selectivity, insufficient efficiency, and safety concerns.^582^ Fang et al. designed a PTP1B-targeting PROTAC based on CRBN and PTP1B inhibitors, displaying remarkable degradation activity by 50-fold compared to inhibitor. Moreover, it reduced the area under the curve of blood glucose from 0 to 2 h to 29% in KM mice, showing promise for long-term antidiabetic therapy.^583^ Besides, PTP1B inhibitors have shown promising effects in other diseases such as cancers,^584^ immunity,^585^ and neurological disorders.^586^ PTP1B degraders are expected to improve treatment for these diseases.

In the treatment of metabolic syndrome, TPDs have shown significant promise. The above studies have demonstrated that TPDs are particularly effective in degrading proteins associated with glucose and lipid metabolism, effectively reversing diseases caused by metabolic abnormalities, such as fatty liver, atherosclerosis, and diabetes. These findings offer new therapeutic strategies that could significantly impact the management of metabolic syndrome. However, the metabolic network in the body is extremely complex, and current research is primarily at the preclinical stage with insufficient data. Future studies are required to further investigate the mechanisms of action of TPDs, confirm their safety and efficacy, and explore their specific applications in clinical treatment. These efforts will help optimize the therapeutic potential of TPDs, providing more precise and effective approaches to managing metabolic syndrome.

### TPD in inflammatory disorders

Autoimmune diseases are characterized by the immune system’s aberrant response against its musculoskeletal system, joints, and peri-articular soft tissues, leading to chronic inflammation and tissue damage. The pathogenesis of these diseases involves complex interactions among genetic, environmental, and immunological factors.^587,588^ Current treatments primarily focus on immunosuppression, which can lead to various side effects and often do not provide a cure.^589^ TPD offers a promising alternative by more selectively and effectively degrading pathogenic proteins that drive autoimmune responses, reducing off-target effects and improving patient outcomes.

#### IRAK4 degraders

In various autoimmune diseases, dysregulated TLR activation has been observed.^590–592^ As previously discussed, IRAK4 plays a pivotal role in the TLR signaling pathway, making it a target of interest for therapeutic intervention. KT-474, an IRAK4 degrader, has been applied in patients with hidradenitis suppurativa (HS) and atopic dermatitis. In phase I clinical trials, KT-474 demonstrated potent and selective degradation efficacy, with an IC50 of 1–2 nM and maximal inhibition reaching 100%. Moreover, KT-474 exhibited excellent safety and tolerability profiles, without TRAEs. It effectively suppressed a wide range of pro-inflammatory cytokines and chemokines, thereby alleviating itching and pain in patients. These promising early results have prompted advancement to phase II trials.^593^ Given IRAK4’s involvement in various other autoimmune diseases,^594^ IRAK4 degraders hold significant potential for broadening therapeutic applications in clinical settings.

#### Janus kinases (JAKs) degraders

JAKs, intracellular non-receptor tyrosine kinases, play a pivotal role in the signaling cascades initiated by various cytokines.^595^ Upon activation, JAKs phosphorylate and dimerize STATs, which subsequently translocate to the nucleus to initiate transcriptional responses regulating immune function, inflammation, and hematopoiesis. Currently, therapeutic strategies targeting JAK2, such as Ruxolitinib and Tofacitinib, have been developed for hematologic malignancies and autoimmune diseases.^596,597^ However, these agents face challenges such as drug resistance and off-target effects, largely due to the high homology within the JAK protein family.^598^ Dual-targeting JAK degraders for JAK1/2 and JAK2/3 have been developed for treating atopic dermatitis, effectively modulating inflammation to reduce disease severity and improve clinical outcomes.^599,600^ In addition, Chang et al. developed multiple JAK-targeting PROTACs based on type I JAK inhibitors, Ruxolitinib and Baricitinib. Notably, compound 8 achieved near-complete degradation of JAK2 without degrading GSPT1, displaying enhanced anti-leukemic efficacy in samples from most patients with acute lymphoblastic leukemia compared to the parent inhibitors.^601^ This innovative approach highlights the potential of TPD as a transformative strategy for therapeutic intervention.

#### HDAC degraders

In addition to its regulatory role in cancer-associated genes, NF-κB stability and DNA binding capabilities are influenced by its acetylation status.^602^ Consequently, targeting HDACs can modulate the acetylation of NF-κB, thereby affecting the expression of inflammatory genes. Cao et al. developed a PROTAC, HD-TAC7, based on class I HDAC inhibitors and pomalidomide, which facilitated the degradation of HDAC3 in RAW 264.7 macrophages stimulated by inflammatory factors. However, the functionality of HD-TAC7 has not yet been validated in specific disease models.^603^

Currently, TPD strategies targeting inflammatory pathways are being developed, offering potential improvements in treating autoimmune diseases. However, further experimental validation is still required. The emerging role of TPD in modulating inflammation in autoimmune diseases is promising, as it provides a novel approach to selectively inhibit pathologically active proteins, potentially leading to more effective and less toxic therapeutic options.

#### BTK degraders

Given BTK’s role in upstream regulation of NF-κB activation,^604^ it has emerged as a therapeutic target for inflammatory diseases. Although several BTK inhibitors have been discovered for the treatment of autoimmune diseases,^605–607^ acquired resistance and toxicities limit their effectiveness.

In 2023, Huang et al. developed a novel class of Ibrutinib-based PROTACs by recruiting CRBN. The most promising compound 15 was able to degrade BTK at low concentration (DC_50_ = 3.18 nM) and reduce the secretion of pro-inflammatory cytokines in lipopolysaccharide-stimulated RAW264.7 cells. Moreover, compound 15 could also suppress inflammatory responses in a mouse model.^608^ This study demonstrated that PROTACs targeting BTK have great potential in the treatment of inflammatory diseases.

#### Stimulator of interferon genes (STING) degraders

The cyclic GMP-AMP STING signaling pathway plays a crucial role in inflammatory response to viral infection and cellular injury.^609^ In this signaling pathway, STING acts as a core modulator to perceive cytosolic cyclic dinucleotides catalytically synthesized by cyclic GMP-AMP synthase,^610,611^ and further stimulate the production of type I interferons and other pro-inflammatory cytokines.^612^ Abnormal activation of STING is intimately tied to many inflammatory syndromes and autoimmune diseases, making it a promising therapeutic target.^613^ Several inhibitors targeting STING have shown great potential as therapeutic agents,^614^ suggesting that PROTACs might serve as an alternative to down-regulating STING-mediated signaling.

In 2022, Liu et al. discovered a series of PROTACs based on small molecular inhibitor C-170 and pomalidomide as CRBN ligand, the most potent compound SP23 was able to degrade STING protein and decrease INFs, IL-6, and CXCL10 levels in monocytic leukemia THP-1 cells. SP23 also exhibited anti-inflammatory and kidney-protective efficacy in a cisplatin-AKI mouse model.^615^ This research provides an example for applying PROTAC technique to develop new anti-inflammatory agents by degrading STING protein.

### TPD in viral infection

Viral infections remain a major public health concern globally, with outbreaks of diseases such as influenza, HIV, and newly emerging viruses like SARS-CoV-2 causing significant morbidity and mortality. Viruses can mutate rapidly, complicating the development of effective vaccines and therapeutic strategies.^616,617^ Current antiviral therapies, including vaccines and small molecule drugs, offer substantial benefits but often fall short in curbing viral resistance and adverse side effects^618,619^ or in providing complete protection. TPD presents a novel therapeutic approach, potentially transforming the landscape of antiviral treatments. By specifically degrading crucial viral proteins, TPD strategies can disrupt the viral lifecycle more effectively than traditional inhibitors, offering a pathway to overcome the limitations of existing therapies.

#### Human immunodeficiency virus (HIV)

HIV continues to pose a significant global public health challenge. While current antiretroviral therapy effectively suppresses HIV replication, it cannot eradicate the virus, necessitating lifelong medication for patients. The Nef protein within HIV critically undermines the host’s immune system, particularly by downregulating major histocompatibility complex class I (MHC-I) and CD4 molecules on T cells, thereby impairing the immune defense against the virus.^620–623^ Research has indicated that Nef inhibitors can counteract this downregulation of MHC-I, enhancing the cytotoxic T lymphocyte response and the clearance of HIV-infected primary lymphocytes. However, SMIs only partially block HIV functions and upon discontinuation, HIV replication resumes. In this context, Smithgall et al. synthesized a Nef-PROTAC that not only effectively reversed the downregulation of MHC-I and CD4 in T cells but also suppressed HIV-1 replication.^624^ Nonetheless, this study has not yet been validated in in vivo models, and further clinical research is needed. This approach opens a promising avenue for potentially curative HIV treatments, highlighting the importance of advancing this innovative technology into clinical trials.

#### Hepatitis B virus (HBV)

HBV remains a significant global health challenge, affecting over 250 million people worldwide.^625^ Although current treatments such as nucleos(t)ide analogs and interferons effectively suppress viral load, they often fail to completely eliminate the covalently closed circular DNA (cccDNA) of the virus, which leads to chronic infection and subsequent liver diseases.^626^ The HBV X protein (HBx) plays a critical role in the viral life cycle by influencing viral replication and modulating host cellular processes.^627,628^ In 2014, Montrose et al. developed PROTACs based on peptides targeting HBx, achieving degradation of both full-length and C-terminally truncated forms of the X protein.^629^ However, this initial effort was preliminary and did not evaluate the impact on HBV replication. Research has shown that the transcriptional activity of cccDNA is subject to extensive epigenetic regulation, opening new avenues for anti-HBV treatment.^630,631^ Guo et al. found that a BRD4 inhibitor significantly inhibited cccDNA transcription through screening of an epigenetic compound library. They further leveraged the BRD4-PROTAC dBET1, which effectively degraded BRD4 and suppressed cccDNA replication.^632^ This strategy may provide an alternative and feasible approach to achieving a functional cure for HBV.

#### Hepatitis C virus (HCV)

HCV is a major global pathogen, infecting over 70 million people worldwide.^633^ While current direct-acting antiviral drugs have significantly improved HCV treatment outcomes, challenges such as treatment resistance persists.^634^ The NS3/4A protease of HCV plays a pivotal role in the viral replication cycle by cleaving and activating viral proteins, thereby evading host immune responses.^635^ Therefore, NS3/4A is considered a promising therapeutic target.^636^ Yang et al. developed DGY-08-097, an NS3/4A degrader based on SMIs, telaprevir, and CRBN ligand, which effectively degraded NS3 protein, including in cells with telaprevir-resistant HCV variants.^637^ DGY-08-097 represents an innovative therapeutic approach that holds promise for overcoming the limitations of current treatments, particularly in addressing drug-resistant viral variants. With further research and clinical trials, these novel treatment strategies are expected to advance into clinical practice, offering more effective and personalized treatment options for patients.

#### Human cytomegalovirus (HCMV)

HCMV is a pervasive pathogen, infecting 60–90% of the adult population worldwide. Although the majority of these infections are asymptomatic, HCMV can cause significant diseases in immunocompromised individuals and newborns. Current antiviral treatments, such as ganciclovir and valganciclovir, target viral DNA replication but often lead to significant side effects and the emergence of drug-resistant strains.^638^ CDKs are crucial for cellular cycle regulation and have been shown to play a role in HCMV replication, positioning them as potential targets for antiviral therapy.^639–641^ Marschall et al. developed a CDK9-directed PROTAC, THAL-SNS-032, which demonstrated 3.7 times higher anti-HCMV activity in HCMV-infected primary human foreskin fibroblasts and a mouse model compared to traditional inhibitors. Moreover, THAL-SNS-032 was also found to inhibit the replication of SARS-CoV-2.^642^ This dual functionality underscores the potential of PROTACs not only in treating HCMV but also in addressing other viral infections.

#### Influenza virus

Influenza remains a pervasive global health threat, leading to significant annual outbreaks that strain healthcare systems and cause substantial morbidity and mortality. While vaccines and antiviral agents like oseltamivir and zanamivir are crucial in managing influenza,^643^ their efficacy can be compromised by rapid viral mutation and the emergence of resistant strains.^644^ Zhou et al.^645^ ingeniously designed a PROTAC based on oseltamivir, which effectively induced the degradation of influenza neuraminidase and demonstrated potent antiviral activity against both wild-type and oseltamivir-resistant strains of the H1N1 virus. Influenza hemagglutinin (HA) that plays a pivotal role in viral entry process via host cell receptor binding and membrane fusion, is an up-and-coming antiviral target. Li et al. synthesized a series of oleanolic acid-based PROTACs as HA degraders. Among them, compound V3 could promote HA degradation at low concentrations and show broad spectrum anti-influenza A virus activity in a 293T cell-based model. This work provides a new direction for the application of PROTACs in potential anti-influenza viral drug discovery.^645^ In addition, Zhao et al. developed a novel PROTAC, named FM-74-103, by leveraging the nucleoprotein (NP) inhibitor nucleozin. FM-74-103 was crafted to inhibit the replication of various viruses, including influenza A virus (IAV), SARS-CoV-2, and CMV. Interestingly, FM-74-103 does not act by directly targeting NP but exerts its antiviral effects through the degradation of GSPT1, a protein previously linked to tumor diseases such as AML.^646^ This discovery not only expands the potential therapeutic applications of GSPT1 degraders but also supports the theory of using GSPT1 as a therapeutic target in these viral infections. The implication that previously reported GSPT1 degraders could be repurposed for treating viral infections opens new avenues for the development of broad-spectrum antiviral therapies, potentially transforming the treatment landscape for multiple viral diseases. In addition to PROTAC degraders, Cen et al. have made significant strides by screening and discovering various microbial metabolites that act as inhibitors of IAV. These metabolites facilitate the ubiquitination and degradation of the IAV endonuclease PA by E3 ligase TRIM25, effectively exerting antiviral effects against both IAV and influenza B virus in vitro and in vivo models, providing a robust antiviral therapy.^645^ Moreover, beyond direct viral replication inhibition, Sun et al. discovered that targeted degradation of cyclophilin A (CypA) can effectively suppress the production of pro-inflammatory cytokines, control cytokine storms, and improve survival rates in mice infected with influenza B virus (IBV).^647^ These approaches, combining direct antiviral and adjunctive therapies, offer new therapeutic strategies for viral infections.

#### Severe acute respiratory syndrome coronavirus 2 (SARS-CoV-2)

Since its emergence in late 2019, SARS-CoV-2, which causes coronavirus disease 2019 (COVID-19), has rapidly evolved into a global pandemic, posing profound challenges to global public health. In the pursuit of effective treatments, TPD technology has shown potential.

Indomethacin (INM), a non-steroidal anti-inflammatory drug, has shown potential as a treatment or adjunct for SARS-CoV-2/COVID-19.^648,649^ Although research has shown that INM can inhibit the replication of SARS-CoV-2, its potency against the virus is limited. To enhance its efficacy, Desantis et al. developed PROTACs based on INM coupled with a VHL ligand targeting PGES-2. These PROTACs demonstrated up to a 4.5-fold improvement in inhibiting viral replication compared to INM alone. In addition, these PROTACs exhibited broad antiviral activity across different genera of coronaviruses.^650^ The main protease (MPro), a highly conserved enzyme among various CoVs, is essential for viral replication and pathogenesis, making it a key target for antiviral drug development. In subsequent research, Desantis et al. used a piperazine-based linker to develop the first class of SARS-CoV-2 Mpro degraders.^651^ In the same year, Alugubelli et al. proposed other Mpro-targeting PROTACs (MPD2) that were effective in inhibiting viral replication, notably including drug-resistant viral variants.^652^ Through these studies, TPD technology has demonstrated significant potential in enhancing the efficacy and expanding the scope of antiviral drugs, offering new directions for future antiviral strategies.

In summary, TPD presents a compelling alternative for antiviral therapy. By targeting and dismantling key components of the viral machinery, TPD enables precise and controlled disruption of viral replication processes, which potentially leads to the development of more effective antiviral treatments. In addition, there is potential to utilize this technology in the development of attenuated vaccines,^653,654^ which could offer a safer and more controlled method of immunization by weakening the virus through specific protein degradation. This evolving application of TPD in viral therapy underscores its potential as a pivotal tool in the development of next-generation antiviral treatments and preventive measures.

### TPD in Down’s syndrome

Down syndrome, characterized by the presence of an extra copy of chromosome 21, is associated with intellectual disability, distinct facial features, and an increased risk of certain medical conditions.^655,656^ Current treatment strategies primarily focus on symptoms management and improving quality of life. Research has shown that removing dysfunctional mitochondria can positively affect cellular function. In 2019, Arimoto et al. developed AUTAC4, a compound that, when applied to human fibroblasts from Down syndrome patients over three days, restored mitochondrial membrane potential and ATP production, and elevated levels of PPARGC1A/PGC-1α, crucial for mitochondrial biogenesis.^242^ More recently, in 2023, Lu et al. introduced ATTEC mT1, composed of GW5074 and a module interacting with the outer mitochondrial membrane protein TSPO. This new formulation induces mitophagy by targeting endogenous TSPO and LC3, offering a novel approach to treating Down syndrome.^657^ These advancements highlight the potential of targeted cellular repair mechanisms in mitigating some effects of genetic disorders.

## New and enabling technologies for TPD

Advancements in technology have significantly expanded the scope of research and application in TPD. These technologies not only facilitate precise control over protein levels but also enhance our understanding of protein functions and their roles in disease mechanisms. As the complexity of protein degradation pathways is unveiled, innovative technologies play a pivotal role in overcoming previous challenges and forging new pathways in drug discovery. This integration of cutting-edge technologies is crucial for pushing the boundaries of current medical science and paving the way for novel therapeutic strategies.

### Computational modeling and artificial intelligence (AI)-aided designs

The development of TPD technologies involves intricate chemical testing and extensive optimization, a process significantly accelerated by application of computational modeling and AI. These technologies streamline the transition from initial screening to final optimization, enhancing drug development efficiency.

For example, high-throughput screening swiftly identifies potential TPD agents, as demonstrated by Guo et al.’s development of the Rapid-TAC platform. This platform utilizes the OPA-amine yielding reaction producing only water as a byproduct.^458^ This reaction achieves high conversion rates and can be performed in parallel, making it a robust tool for the rapid development of PROTACs featuring diverse linkers. This process is further facilitated by a multi-component reaction platform.^658^ The integration of computational tools, including 3-D modeling and deep learning, automates the design of novel PROTACs on platforms like PROTACable.^659^ These tools are crucial in structuring molecular docking^660^ and dynamics simulations,^661^ which delve into protein-ligand interactions. They provide insights into how molecular modifications affect the stability and efficacy of PROTACs. In addition, neural networks^662–665^ and other AI-driven methodologies process extensive datasets to predict drug responses and refine molecular designs more efficiently than traditional methods, identifying subtle chemical structure changes that significantly impact drug efficacy and resistance. The synergy of DNA-encoded libraries,^657,666,667^ solid phase synthesis,^668–670^ and direct-to-biology^671^ techniques facilitates extensive drug screening and optimization, further streamlining the drug development pipeline. These advancements ensure that TPD agents are developed rapidly, effectively and safely, promising substantial progress in therapeutic innovations.

### Delivery technologies

**Fig. 7:**
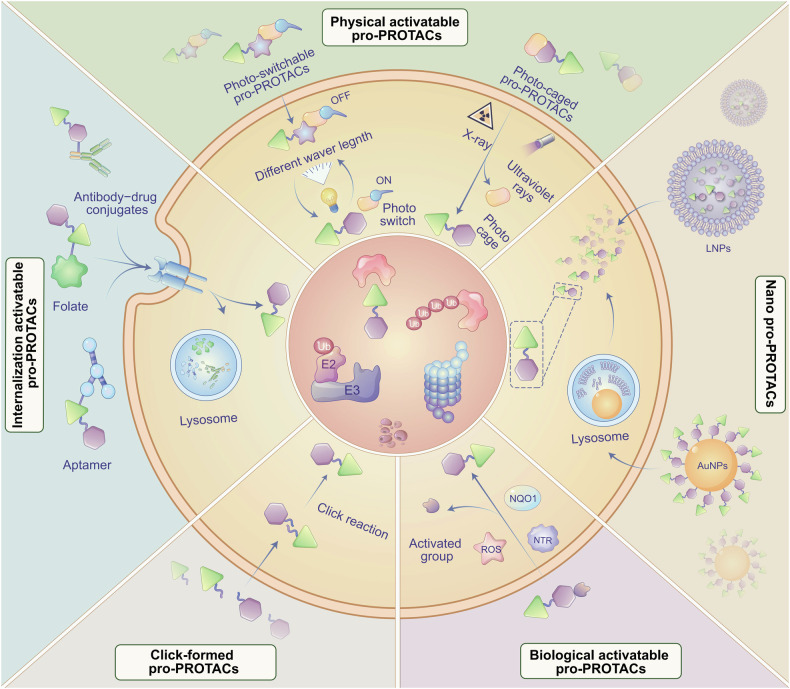
The novel delivery technologies for TPD. Click-formed pro-PROTACs with two precursors: a tetrazine-tagged E3 ligase ligand and a trans-cyclooctene-functionalized POI ligand, can be assembled into a PROTAC in the cytosol. Internalization-activatable pro-PROTACs with specific ligand groups (aptamer, folate, or antibody–drug conjugates) attached can bind to cell surface receptors. After undergoing lysosomal processing, PROTACs are released to function. Physical-activatable pro-PROTACs function under the control of light. Upon light irradiation (X-ray or ultraviolet rays), the photo-caging group is cleaved, rendering PROTACs active irreversibly. On the other hand, with light irradiation at particular wavelengths, photo-switchable PROTACs can reversibly transform into isomeric forms to mediate therapeutic action. After AuNPs pro-PROTACs and LPs pro-PROTACs enrich PROTACs into the cell, they undergo lysosomal processing, which releases the PROTACs to function effectively. Biological-activatable pro-PROTACs can be activated by the special microenvironment of tumors, such as high levels of ROS. PDT Photodynamic therapies, NTR nitroreductase, NQO1 NAD(P)H quinone dehydrogenase 1 enzyme, ROS reactive oxygen species

The effectiveness of large molecule drugs is often limited by their poor cell membrane permeability. In addition to structural modifications of TPDs to enhance this, ongoing research focuses on developing innovative formulations and delivery mechanisms (Fig. 7). These efforts aim to reduce non-specific interactions with biological systems, minimize side effects by avoiding unnecessary tissue accumulation, and ensure controlled release at targeted disease sites, both spatially and temporally.

#### Click-formed pro-PROTACs (CLIPTACs)

To address the poor solubility and cell permeability of PROTACs due to their high molecular weight, Lebraud et al. introduced CLIPTACs. They are constructed from two precursors: a tetrazine-tagged E3 ligase ligand and a trans-cyclooctene-functionalized POI ligand.^672^ These precursors can be assembled into a PROTAC in the cytosol once entering the cells without a chemical catalyst. CLIPTAC has been successfully utilized in designing PROTAC that target transcriptional factors, such as NF-κB and Drosophila E2 factor, through the “click” reaction.^12^ This approach shows promise in resolving the physicochemical property issues of PROTACs.

#### Internalization activatable pro-PROTACs

Classic antibody–drug conjugates are well-established therapeutic agents, exhibiting substantial antitumor efficacy, along with stability and favorable pharmacokinetic profiles.^673^ Inspired by the success of antibody–drug conjugates, internalization activatable pro-PROTACs have been developed. Pillow et al.^459^ synthesized the first degrader–antibody conjugate CLL1-1, via linking GNE-987 to a CLL1-targeting antibody. The pharmacokinetic profile of CLL1-1 was remarkably improved and exhibited greater anti-tumor effects in mice than GNE-987. These findings highlight the promise of degrader–antibody conjugates as a therapy for the tumor-specific degradation of POIs.

Aptamers, which are short single-stranded oligonucleotides, can be easily modified as molecular antibodies, and delivered to specific targets. He et al. developed the first aptamer–PROTAC conjugate by linking AS1411, aptamer-modified liposome, with MZ1, a BRD4 PROTAC. This innovative approach resulted in cellular uptake and internalization of the APC, ultimately leading to the specific degradation of the target protein.^674^

While folate receptor α (FOLR1) is usually overexpressed in many solid tumor cells, it exhibits minimal expression in normal cells. Thus, binding to FOLR1 is recognized as an effective route for cancer-specific targeting. E3 ligase ligand with an attached folate group was used to construct the PROTAC. Studies showed that such PROTAC degrades targeted proteins in a FOLR1-dependent manner in vitro.^404^ However, adding additional protein binding moiety to a PROTAC inevitably increases its molecular weight. Further investigations are needed to improve drug properties of folate-PROTACs while maintaining the selectivity toward cancer cells.

#### Physical activatable pro-PROTACs

To enhance spatiotemporal precision and control off-target toxicity, the utilization of an external physical stimulus to regulate the activity of PROTACs would be beneficial. Photodynamic therapies have been integrated into PROTACs, resulting in photo-caged and photo-switchable PROTACs. Photo-caged PROTACs feature ligands with a photo-caging group, which masks their activity until exposed to light. Upon light irradiation, the photo-caging group is cleaved, rendering PROTACs active and leading to irreversible degradation.^318,675,676^

On the other hand, the photo-switchable PROTACs recruited a photo-switch to the linker, thereby achieving reversible photo-response. With light irradiation at particular wave-lengths, photo-switchable PROTACs can efficiently transform isomeric forms to mediate therapeutic action. Some studies have validated the reversibility of photo-switchable PROTACs.^298,457,677^ It is worth noting that current light-responsive PROTACs primarily rely on ultraviolet rays, limiting their application to superficial tumors, such as hematological and skin tumors. X-ray radiation, with its deep tissue penetration, provides an alternative approach. Yang et al. synthesized a radiotherapy-triggered PROTACs (RT-PROTACs), which become active upon exposure to X-ray radiation. These RT-PROTACs effectively degraded target proteins and exhibited a synergistic antitumor effect when combined with X-rays in xenograft models.^678^

Subsequently, two nanoparticle-based PROTACs combined with near-infrared light have been developed. These innovations overcome the limitations associated with short-wavelength light-controlled PROTACs, providing a promising strategy for safer and more effective applications in deeper tissues.^679,680^ These advancements offer a promising strategy for achieving spatiotemporal release of active PROTAC to control protein degradation. However, there is an urgent need to optimize near-infrared irradiation-responsive groups to ensure compatibility with safer and more deeply penetrating applications.

#### Biological activatable pro-PROTACs

Apart from external physical stimuli, inherent features of the tumor microenvironment can also be harnessed to enhance the selectivity and decrease off-target effects of PROTACs. Solid tumors are known for their hypoxic microenvironment, along with elevated levels of nitroreductase, NAD(P)H quinone dehydrogenase 1 (NQO1) enzyme, and reactive oxygen species (ROS),^681,682^ all of which can potentially be used for designing pro-PROTACs to achieve desired control of PROTACs activation.

One approach involves introducing a hypoxia-activated group onto the ligands of POI or E3 ligase, resulting in the creation of hypoxia-activatable pro-PROTACs (ha PROTACs). Under hypoxic conditions, the added cage group chemically reacts with nitroreductase and is removed to release active PROTACs in tumor tissues.^674,683^ Ha PROTACs demonstrated spatially antitumor activity, as confirmed in both in vitro and in vivo studies. Given the differences in ROS levels between tumor and normal tissues, Chen’s group grafted an arylboronic acid group onto the CRBN ligand and obtained a ROS-responsive PROTAC.^684^ After the boronic acid group is cleaved by H2O2, the restored PROTACs specifically degrade proteins of interest (POIs) in tumors in a dose-dependent and time-dependent manner. NQO1 enzyme is often overexpressed in tumor cells and can catalyze the reduction of β-lapachone, generating plentiful ROS.^685^

Wang et al. introduced a trimethyl-locked quinone group sensitive to NQO1 into a BRD4-targeting PROTAC, masking its activity until NQO1 is present in tumor cells, which cleaves the quinone group and restores degradation capability. They also developed a ROS-triggered PROTAC, enhancing its degradation activity and cell selectivity in the presence of NQO1 and β-Lap.^686^ While the approach by harnessing the specific biology of the tumor environment to provide better selectivity is promising, it is important to note that further investigations are needed to extend the utility of the approach for therapy development due to the heterogeneity of tumors.

#### Nano pro-PROTACs

With small particle size, nanoparticles (NPs) can traverse vessels and bypass tissue barriers, which allows them to passively accumulate in tumor systems. Due to their high specificity, enhanced permeability, controlled drug release, and capability to carry larger drug payloads, NPs are increasingly utilized in cancer treatment^687^ and TPD delivery systems. Gold nanoparticles have been developed for multi-headed PROTACs aimed at treating NSCLC, demonstrating superior ALK degradation and tumor-specific accumulation compared to traditional dual-functional PROTACs. However, pharmacokinetics in vivo were not assessed.^688^ In addition, Li et al. innovatively proposed a lipid-based split-and-mix nano self-regulating platform, LipoSM-PROTAC, which significantly degrades ERα protein in vitro at lower drug concentrations compared to peptide-based SM-PROTAC. Moreover, this system also incorporates folic acid, achieving precise targeting.^689^ The combination of nano PROTACs with chemical groups that responded to tumor microenvironment further increased spatiotemporal controlled targeted degradation.^680,687^

Peptide-based PROTACs characterized by their high affinity and inherent good safety profile, are nevertheless hindered by limited permeability, which is expected to be overcome with the integration into nanomedicine. Semiconducting polymer nano PROTACs have been synthesized to degrade immune-related proteins, thereby reprogramming tumor microenvironment and curbing tumor growth.^690,691^ Although it is promising to develop the nano PROTACs as therapies, a lot of work is still needed due to challenges like complex synthesis processes and unknown drug safety as a new modality.

Recent studies have focused on enhancing the spatiotemporal control and selectivity of PROTACs through novel formulations and delivery techniques, yet there is a need for a more in-depth discussion on the advantages, disadvantages, and development prospects of each formulation and delivery method. In Table 6, we compare the potential of each delivery technology, revealing the technical intricacies and future possibilities that could revolutionize cancer treatment. The advancements in computational models, drug property enhancement, and delivery technology collectively contribute to drug development for TPD, thereby improving therapeutic outcomes and safety.

**Table 6:** The features of novel delivery systems

Technology	Advantages	Limitations	Potential applications	Future prospects
CLIPTAC	Intracellular assembly bypassing solubility and permeability issues	Potential extracellular reactions	Biological barriers such as the blood-brain barrier	Optimize reaction rate, improve click-release strategy, and enhance tissue selectivity
Internalization activatable pro-PROTAC	Enhanced pharmacokinetics and tumor specificity	Synthetic complexity	Tumors with specific cancer markers highly expressed	Expand to more cancer types with specific overexpressed markers
Physical activatable pro-PROTAC	Enhanced spatiotemporal precision via controlled activation	Restricting use in deep tumors	Superficial tumors	Developing PROTACs with enhanced tissue-penetrating photo-caging group
Biological activatable pro-PROTAC	Higher selectivity due to activation by tumor-specific environmental conditions	Effacacy limitation due to variable tumor environments	Heterogeneous solid tumors, especially in low-oxygen or high-ROS environments.	Precisely control activation conditions to match the microenvironment characteristics of different tumor types
Nano pro-PROTAC	Enhanced permeability and tumor accumulation, larger drug payloads and precise targeting with environmental responsiveness	Challenges in synthesis and drug safety profiles	Solid tumors and hard-to-penetrate tumor tissues	Resolve stability and safety issues of nanoparticles in production and in vivo applications

## Challenges in TPD

Despite the above inspiring effects and enormous potential of TPD, the road to clinical application is paved with substantial challenges. These challenges span from the design and development of effective degraders to addressing safety concerns. Many CRBN-binding molecules are low molecular weight compounds with favorable drug properties. However, a major challenge in MGs discovery is the lack of rational design approaches. This is primarily due to the dynamic nature of CRBN-Glue-Target interactions. Advances in technology or methodology are essential to enhance the discovery of new MGs. While PROTACs can be designed with precision, their drug-like properties are greatly influenced by the composition of TPD molecules. Contrary to the rule-of-five, PROTACs’ large molecular weight and complex structure often result in poor water solubility, high first-pass metabolism, challenges in penetrating cell membranes, and difficulties with oral administration.^692^ Despite these challenges, PROTAC design is transforming the paradigms of small molecule drug discovery. Advancements require a careful balance between efficacy and bioavailability, particularly in optimizing stability and solubility. The length, rigidity, and stereoselectivity of the linker are crucial for enhancing affinity and pharmacokinetic properties. Advances such as the development of AlphaFold^693^ have enhanced the efficiency of TPD design by facilitating the analysis of ternary complex structures and PPIs, providing insights into the tractability and stability of ligands for targets and linking enzymes. Lysosome-based degradation approaches have broadened the scope of protein degradation, enabling the breakdown of extracellular proteins.^136^ However, their application across various therapeutic areas and the understanding of their mechanisms are still in preliminary stages. Autophagy, highly conserved in eukaryotic cells and active in all mammalian cell types, poses a higher risk of off-target toxicity. Current challenges include ensuring the stability and targeted delivery of these chimeric molecules, mitigating potential off-target effects, and comprehending the long-term implications of manipulating lysosomal pathways. Moreover, the variability in E3 ligase activity across different tissues^78^ complicates the predictability and effectiveness of TPD. Resistance emergence due to genetic alterations in key E3 ligase genes like VHL and CRBN further complicates long-term usage.^694–696^ Exploring low-toxicity ligands, such as BIRC2 and RNF114,^697,698^ offers a viable path forward by leveraging novel E3 ligases in therapeutic applications. Han et al.‘s innovative approach of utilizing big data analytics to identify potential E3 ligases for PROTAC interaction^699^ exemplifies the potential to expand the scope of targeted degradation. As TPD technology continues to evolve, integrating more E3 ligases and enhancing precision, efficacy, and safety is anticipated, potentially elevating the therapeutic impact of this groundbreaking approach. Addressing these critical factors is essential for advancing the clinical applications of TPD.

The unique advantage of TPD lies in its potent protein degradation capabilities. However, off-target effects can also introduce severe toxicity. Current challenges include ensuring the stability and delivery of these chimeric molecules to the desired tissues, mitigating potential off-target effects, and understanding the long-term implications of manipulating the lysosomal pathways. There is a crucial need for detailed structural information, highly selective warheads with increased affinity, further SAR data, and precise modeling supported by experimental evidence. Utilizing tissue-selective ligases can aid in treating tissue-specific diseases,^78^ while other targeting strategies, such as light-induced PROTACs^700^ and tissue-specific antibody-mediated PROTACs,^404^ enhance the specificity and safety of PROTAC delivery. However, unfavorable physicochemical properties may restrict cellular permeability and bioavailability.

Due to the prolonged action and recyclability of TPD, its pharmacodynamics and pharmacokinetics differ from traditional SMIs. It is crucial to timely monitor in vivo drug concentrations, guiding the selection of effective doses for human clinical use. This monitoring helps prevent off-target effects and inhibition of drug efficacy caused by high concentrations of PROTACs, which can lead to the hook effect. Stable isotope labeling by amino acids in cell culture (SILAC) mass spectrometry and pulsed SILAC approaches are instrumental in determining the protein turnover rates.^701^ These methods support PK-PD modeling,^702^ facilitating the systematic and rational design of TPD to achieve the necessary efficacy while maintaining sufficient target selectivity to minimize toxicity.

AI and machine learning are revolutionizing drug design by bringing unprecedented precision, which not only accelerates the discovery of novel degraders but also optimizes pharmacokinetic profiles and predicts therapeutic outcomes. Despite these advancements, challenges such as data inadequacy and high computational demands persist.^378^ The multidisciplinary approach that integrates advanced computational models, structural biology, biochemical strategies, and medicinal chemistry can significantly enhance the design and delivery of these molecules. Leveraging such diverse technologies helps address current limitations and drives the development of effective and precise therapeutic strategies.

## Conclusions and perspectives

The evolution of TPD marks a revolutionary milestone in the therapeutic landscape, notably enhancing the treatment of diseases through innovative modalities such as PROTACs and MGs. Recent advancements in lysosome-based degradation technologies have broadened the spectrum of strategies for targeting disease-associated proteins. Unlike SMIs, TPDs exploit the body’s intrinsic protein degradation systems to selectively target and degrade disease-causing proteins. This approach minimizes off-target effects, circumvents resistance due to target mutations, and can target proteins previously considered “undruggable.” Collectively, these characteristics significantly contribute to the development of safer and more effective treatments.

Currently, TPDs have demonstrated significant therapeutic effects in various fields, such as neurodegenerative diseases,^703^ autoimmune disorders,^603^ cardiovascular disease,^704^ and infectious diseases.^637^ As the earliest type of TPDs, MGs like lenalidomide and pomalidomide have achieved clinical success in MM and MDS, sparking significant interest in developing new MGs and PROTACs. CC-92480, an advanced IMiD, is designed to overcome the drug resistance observed with lenalidomide and pomalidomide.^269^ Clinical trials indicate its potential approval for MM and T-cell lymphomas resistant to existing therapies within the next 1–2 years. Several MGs targeting IKZF1/3 degradation, similar to CC-92480, are currently under development at various stages. In addition, MGs targeting proteins such as GSPT1^490^ and RBM39^131^ are currently in clinical trials, highlighting the wide-ranging potential of CRBN-binding MGs. Compared to MGs, PROTACs benefit from more rational design approaches, drawing on extensive experience accumulated during their development. Numerous PROTACs are currently in clinical trials, including ARV-471, which is in an ongoing Phase III trial. Enhancing bioavailability remains a critical challenge in PROTAC design, necessitating substantial medicinal chemistry efforts for optimization. In contrast, lysosomal degradation technologies, though less explored, offer novel strategies for addressing traditionally undruggable targets by degrading aggregated proteins, damaged mitochondria, and pathogens.^136^ These have shown promising degradation capabilities, particularly in treating neurodegenerative and genetic disorders. Their application requires specialized design strategies and further research to optimize selective protein degradation via lysosomes. Future clinical trials are likely to expand on lysosome-targeted TPDs. Given the clinical success of IMiDs, new MGs and PROTACs are being tested in combination with established therapies, including immuno-oncology treatments, targeted SMIs, and traditional chemotherapeutics. Many of these trials involve combination therapies, addressing complex diseases such as melanoma, which often features BRAF mutations and PTEN loss.^705,706^ Although PROTACs demonstrated high specificity, disease progression can still occur due to incomplete pathway inhibition or emerging drug resistance. Combining PROTACs with other therapeutic approaches is intended to produce synergistic effects and enhance overall treatment efficacy.

TPD offers significant potential in drug development, with active efforts to refine its design and broaden its applications. These efforts include exploring new E3 ligases, identifying novel targets, establishing design principles, and enhancing delivery methods. Despite its promise, challenges such as incomplete pathway inhibition and drug resistance remain. Overcoming these obstacles requires collaborative efforts between academia and industry, focused on theoretical advancements, clinical trial validation, and widespread clinical adoption.

As technologies advance, and identification of degradable substrates becomes more precise, TPD is poised to facilitate profound degradation of specific proteins, positioning itself as an expedient and user-friendly alternative to other new technologies such as CRISPR-Cas9. This approach not only allows for precise protein level control through its reversibility—simply by stopping the administration of the degrader—but also offers enhanced specificity, thereby reducing off-target effects.^707^ Such features significantly accelerate basic research, owing to TPD’s rapid action and straightforward application.

In conclusion, TPD represents a pivotal shift in drug development paradigms. Ongoing research and clinical trials will undoubtedly provide deeper insights. As the full potential of these innovative molecules is explored, TPDs are poised to become a cornerstone of next-generation therapeutic strategies, providing more effective and personalized treatments for human diseases.
